# A strategy to discover new organizers identifies a putative heart organizer

**DOI:** 10.1038/ncomms12656

**Published:** 2016-08-25

**Authors:** Claire Anderson, Mohsin A. F. Khan, Frances Wong, Tatiana Solovieva, Nidia M. M. Oliveira, Richard A. Baldock, Cheryll Tickle, Dave W. Burt, Claudio D. Stern

**Affiliations:** 1 Department of Cell and Developmental Biology, University College London, Gower Street, London WC1E 6BT, UK; 2 Department of Genomics and Genetics, The Roslin Institute, University of Edinburgh, Easter Bush, Midlothian, EH25 9RG Scotland, UK; 3 Biomedical Systems Analysis Section, MRC Human Genetics Unit, IGMM, University of Edinburgh, Crewe Road, Edinburgh, EH4 2XU, UK; 4 Department of Biology & Biochemistry, University of Bath, Bath BA2 7AY, UK

## Abstract

Organizers are regions of the embryo that can both induce new fates and impart pattern on other regions. So far, surprisingly few organizers have been discovered, considering the number of patterned tissue types generated during development. This may be because their discovery has relied on transplantation and ablation experiments. Here we describe a new approach, using chick embryos, to discover organizers based on a common gene expression signature, and use it to uncover the anterior intestinal portal (AIP) endoderm as a putative heart organizer. We show that the AIP can induce cardiac identity from non-cardiac mesoderm and that it can pattern this by specifying ventricular and suppressing atrial regional identity. We also uncover some of the signals responsible. The method holds promise as a tool to discover other novel organizers acting during development.

## Abstract (web summary)

Organizers are regions in the embryo that induce cell fate and impart pattern on neighbouring regions. Here, the authors search for new organizers based on a common gene signature, and show that the Anterior Intestinal Portal endoderm induces cardiac identity, specifies ventricle and inhibits atrial character.

## Graphical abstract

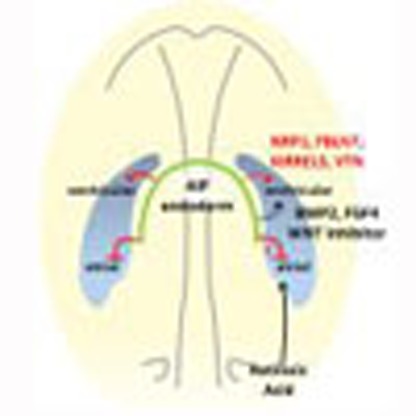

Organizers are formally defined as signalling regions, unique in being able both to induce and to pattern adjacent tissue^1^. The dorsal lip of the blastopore, which can induce a complete secondary axis in amphibian embryos, was the first organizer to be discovered, in 1924 (ref. 2). Given the complexity of the vertebrate embryo, one might expect that many organizers should exist, but only a few others have been described^3^: Hensen’s node (the amniote equivalent of the dorsal lip of the blastopore, which can induce and pattern the central nervous system)^4^, the notochord/floor-plate (which can induce and organize different sets of neurons in the neural tube)^5^, the zone of polarizing activity (ZPA, which can induce a patterned set of limb elements)^6^ and the midbrain–hindbrain boundary (isthmus, which can specify and pattern the adjacent regions of the midbrain/tectum and hindbrain/cerebellum)^7^. One reason why so few organizers have been found could be that their discovery requires grafting appropriate tissues at the right time and place, and there are too many possible combinations.

It has been shown that in some cases organizers can substitute for each other. Most strikingly, a graft of Hensen’s node from an early (primitive streak stage) embryo into the anterior limb bud of a much later embryo can mimic the action of the limb organizer, the ZPA, by inducing and patterning a full set of skeletal elements including the digits^8^. This raises the intriguing possibility that organizers may share a genetic signature (‘synexpression’) that confers them with inducing and patterning properties. If this is the case, we should be able to use this genetic signature to point to potential new organizing regions. In this study, we tested this idea by comparing the transcriptomes of three known amniote organizers (Hensen’s node, the notochord/floor-plate and the ZPA). This defines a synexpression set of 48 transcripts that are either enriched or depleted in organizers, which we then used to explore the embryo for other regions of synexpression. This suggested that the endoderm of the anterior intestinal portal (AIP) might be a new organizer. The AIP is an endodermal invagination appearing with the head-fold, which moves caudally down the embryo to form the foregut^9^. As it does so, it is closely associated with the developing heart tube. We therefore performed a series of experiments to test whether the AIP can act as an organizer of the heart. Ablation and transplantation experiments *in vivo*, along with co-culture *in vitro*, revealed that the AIP endoderm can induce heart fate from non-cardiac mesoderm as well as pattern the heart field by specifying ventricular and suppressing atrial regional identity.

## Results

### Defining a gene signature for organizers

**Figure 1:**
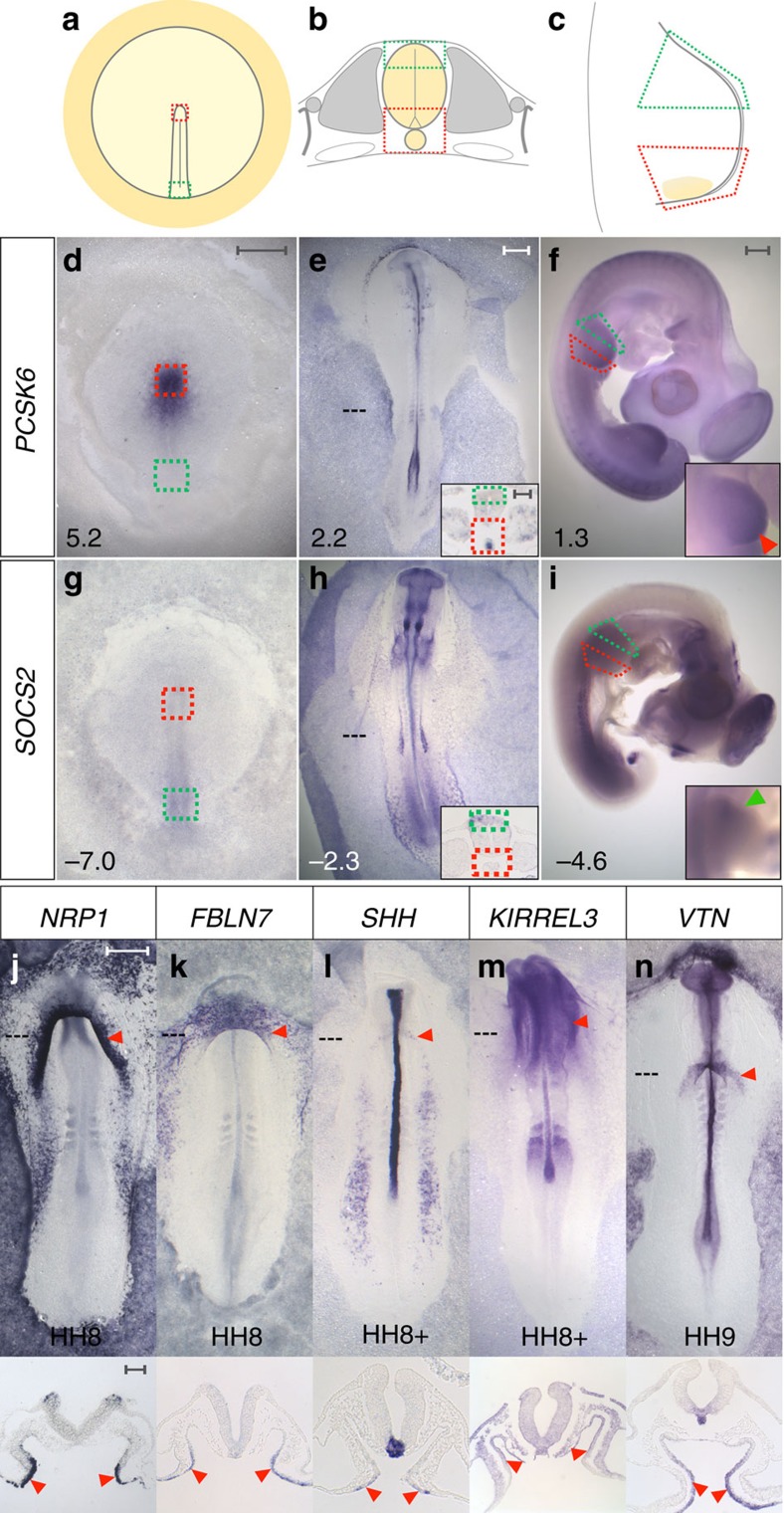
Transcriptome comparison of known organizers reveals genes co-expressed in organizers and in the Anterior Intestinal Portal endoderm. (**a**–**c**) Experimental design. (**a**) Hensen’s node (red box) was compared with posterior primitive streak (green box); (**b**) notochord and ventral neural tube (red box) was compared with dorsal neural tube (green box) and (**c**) posterior wing bud (red box) was compared with anterior wing bud (green box). (**d**–**i**) One gene enriched in organizers is *PCSK6* (**d**–**f**, red boxes, red arrowhead in **f**-inset) and *SOCS2* exemplifies a depleted gene (**g**–**i**, green boxes, green arrowhead in **i**-inset). Fold-change values are indicated. (**j**–**n**) Examples of organizer-enriched transcripts expressed in the anterior intestinal portal endoderm (AIP) (red arrowheads): *NRP1* (**j**), *FBLN7* (**k**), *SHH* (**l**), *KIRREL3* (**m**) and *VTN* (**n**). Scale bars, 0.5 mm in whole-mounts and 0.1 mm in sections.

To investigate whether organizers have a common genetic signature of enriched and depleted transcripts, we designed a differential microarray screen in chick embryos, comparing three known organizers to their most similar non-organizer tissue. First, we chose stage (HH^10^) 3^+^/4 Hensen’s node, which induces and patterns the neural plate^11^, compared with the posterior primitive streak, which cannot (Fig. 1a). We also sampled the later node (HH6), which has lost its ability to induce a full neural plate^11^. This three-way comparison was designed to reveal transcripts associated with full organizer function. Second, we selected the notochord and floor-plate at HH10-11, responsible for organizing the dorso-ventral axis of the neural tube^5^, and compared this to the dorsal neural tube (Fig. 1b). Finally, we compared the posterior wing bud, containing the ZPA^6^, to the anterior wing bud at HH20-21 and HH24 (Fig. 1c). Each comparison generated a list of transcripts significantly enriched or depleted in that organizer (≥1.2-fold the log_2_ of the expression level, and a false discovery rate ≤0.05. The dataset was submitted to ArrayExpress with the title ‘Microarray analysis of chick embryonic tissues: gastrulation, neural tube/notochord and limb development’ and given accession number E-MTAB-4048). These lists were then combined using a Boolean algorithm to find enriched or depleted transcripts common to all three organizers. This approach uncovers relative changes in expression between organizer cells and neighbouring regions, even if the absolute level of expression is widely different at different stages: a putative organizer gene set of 31 enriched and 17 depleted transcripts is revealed (Supplementary Fig. 1a–d and Supplementary Data 1). Interestingly, over 60% of the enriched genes encode membrane-associated or secreted molecules and 7% represent transcription factors, whereas transcription factors are much more frequent (38%) among the depleted genes (Supplementary Fig. 1c). One possible interpretation of this is that the synexpression set is enriched for signalling molecules emitted by the organizer and depleted of transcriptional repressors that suppress the organizer state.

### The AIP endoderm as a candidate organizer

Next, we used this set to verify the expression of the selected genes (Fig. 1d–i, Supplementary Figs 2 and 3), as well as to explore whether the synexpression signature occurs in other places and stages during development. A large-scale *in situ* hybridization analysis of the 48 genes was undertaken from pre-primitive streak stage up to HH27; these expression patterns can be browsed on eChick atlas^12^ (www.echickatlas.org). One embryonic region appropriately expresses 35 genes from the organizer gene set: the endoderm of the AIP. At some stage between HH7-14, 20/31 enriched transcripts are detected (Supplementary Fig. 4a–t), 15/17 organizer-depleted genes are appropriately absent from the early AIP (Supplementary Fig. 4u–k), and SOCS2 and BTG2 are absent before HH10 and HH14 respectively (Supplementary Fig. 4l′,m′). Thus, the AIP shares a similar transcriptional profile to other known organizers.

There is substantial evidence that the early endoderm adjacent to the bilateral cardiac mesoderm is required for normal heart formation in chick, amphibian and fish embryos^13^,^14^, but less is known about the AIP endoderm of later stages. Genetic or manual ablation of the AIP endoderm in mouse and chick embryos results in cardia bifida^15^,^16^,^17^,^18^,^19^ (failure of the bilateral heart progenitors to fuse in the midline), rotation defects^16^ and downregulation of early cardiac genes^15^, indicating that a functional AIP endoderm is required for normal heart formation, but its inducing and patterning abilities have not been tested. Could the AIP be an organizer of the heart?

### The AIP can induce cardiac identity from non-cardiac mesoderm

**Figure 2:**
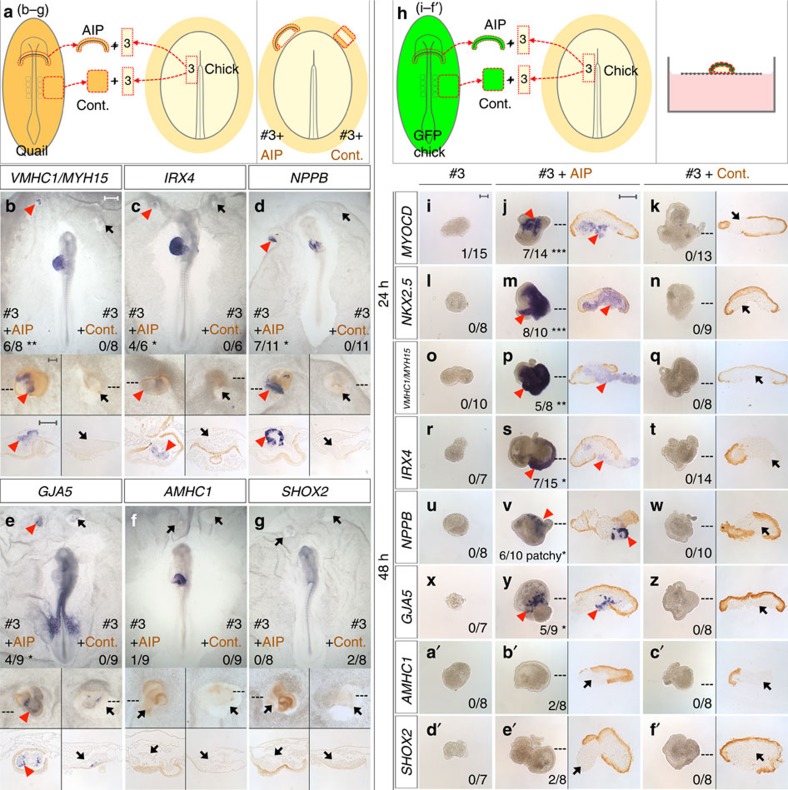
AIP induces cardiac and ventricular identity in non-cardiac mesoderm. (**a**) AIP or control (Cont.), lateral endoderm from a HH8 quail co-cultured with anterior-medial mesoderm (#3) from a HH5 chick embryo overnight in the anterior area opaca of a host chick. (**b**–**g**) Regional myocardium markers *VMHC1/MYH15* (**b**), *IRX4* (**c**), *NPPB* (**d**) and *GJA5* (**e**) are induced in #3-mesoderm by quail (brown) AIP (red arrowheads), but not by control endoderm (Cont., black arrows), whereas *AMHC1* (**f**) and *SHOX2* (**g**) are not induced (black arrows). (**h**) *In vitro* co-culture of #3-mesoderm with GFP-AIP (**j**,**m**,**p**,**s**,**v**,**y**,**b’**,**e’**) or -Cont. (**k**,**n**,**q**,**t**,**w**,**z**,**c’**,**f’**) for 24 (**j**,**k**,**m**,**n**) or 48 h (**p**,**q**,**s**,**t**,**v**,**w**,**y**,**z**,**b’**,**c’**,**e’**,**f’**). No expression is seen in #3-mesoderm cultured alone (**i**,**l**,**o**,**r**,**u**,**x**,**a’**,**d’**). AIP (brown) induces *MYOCD* (**j**), *NKX2.5* (**m**), *VMHC1/MYH15* (**p**), *IRX4* (**s**), *NPPB* (**v**) and *GJA5* (**y**) in #3-mesoderm (red arrowheads), but Cont. does not (brown, **k**,**n**,**q**,**t**,**w**,**z**, black arrows). Neither *AMHC1* nor SHOX2 are induced (black arrows) by AIP (**b’**,**e’**) or by Cont. (**c’**,**f’**). *P≤0.05, **P≤0.005, ***P≤0.0005 using two-tailed Fisher's exact test. Scale bars, 0.5 mm in whole-mounts and 0.1 mm in insets, explants and sections.

We first confirmed that ablation of the AIP at HH8 does indeed cause cardia bifida and abnormal heart rotation (Supplementary Fig. 5). Next, to test if the AIP can induce cardiac identity, a necessary prerequisite is to identify a suitable responding tissue. Fate and specification maps indicate that the paraxial mesoderm adjacent to Hensen’s node at HH5 (prospective head mesoderm) is neither fated nor specified as heart^20^,^21^,^22^,^23^,^24^, but is competent to respond to cardiac inducing signals^22^. We compared this mesoderm (named #3) with four other regions of HH5 mesoderm (Supplementary Figs 6a and 7a): #3 does not contribute to the heart when grafted homotopically (Supplementary Fig. 7b–h), and is the only region of mesoderm that does not express cardiac markers when explanted either *in vitro* (Fig. 2 i,l,o,r,u,x,a′ and Supplementary Table 1) or in a host embryo (Supplementary Fig. 7j–v and Supplementary Table 2). Mesoderm #3 is therefore suitable for testing whether the early AIP can induce heart fate. The AIP induces early cardiac markers, *MYOCD*, *NKX2.5* (Fig. 2j,m and Supplementary Fig. 8b,c), *GATA4*, *TBX5*, *ISL1* and *MEF2C* in explants of #3-mesoderm (Supplementary Fig. 8d–g), while co-culture with control (Cont.), non-AIP endoderm from the lateral embryo, largely does not (Fig. 2k,n, Supplementary Fig. 8b–h, Supplementary Tables 2 and 3). Strikingly, spontaneous beating is observed after 48 h of *in vitro* co-culture of #3-mesoderm with AIP (Supplementary Movie 1), but not with control, non-AIP endoderm. Together these results show that the AIP endoderm can induce cardiac identity in mesoderm that is not otherwise fated to become part of the heart.

### Patterning by inducing ventricular identity and suppressing atrial character

Many of the known early heart markers are normally expressed in the bilateral cardiac mesoderm before the AIP forms (around stage HH5; Supplementary Fig. 6a–f), whereas regional markers of anterior–posterior heart tube patterning are observed later (after HH9; Supplementary Fig. 6v–n′). Ventricular markers (*VMHC1*/*MYH15*, *IRX4* and *NPPB*) are initially expressed in the medial splanchnic mesoderm^25^,^26^,^27^ immediately adjacent to the AIP (Supplementary Fig. 6d′,f′,g′), and the atrial marker *AMHC1* is in the lateral splanchnic mesoderm^28^ (Supplementary Fig. 6c′). In #3-mesoderm explants cultured either in a host embryo or *in vitro*, AIP induces *VMHC1/MYH15*, *IRX4* and patchy *NPPB* expression (Fig. 2b–d,p,s,v, Supplementary Tables 2 and 3). AIP also induces *GJA5* (Fig. 2e,y), which marks the anterior ventricular myocardium, endocardium and outflow tract (Supplementary Fig. 6h′). AIP does not induce *AMHC1* (Fig. 2f,b′) or *SHOX2* (Fig. 2g,e′), a marker of the sinoatrial node (Supplementary Fig. 6i′,p′). These results show that the AIP can induce ventricular cardiac identity in mesoderm not destined to this fate.

Previous tissue recombination experiments using HH5-6 chick explants have demonstrated that anterior-lateral endoderm adjacent to the early cardiogenic mesoderm can induce heart gene expression in posterior primitive streak^29^. Are the induction properties of the HH8 AIP endoderm distinct to the early anterior-lateral endoderm? To test this, we co-cultured HH5 anterior-lateral endoderm with non-cardiac #3-mesoderm (Supplementary Fig. 9a and Supplementary Table 4). Unlike AIP endoderm, anterior-lateral endoderm does not induce ventricular markers (Supplementary Fig. 9b–e) nor does it induce *AMHC1* (Supplementary Fig. 9f). These data show that the anterior-lateral endoderm is different to the AIP endoderm and that the ability to induce ventricular character is a property specific to the AIP endoderm.

**Figure 3:**
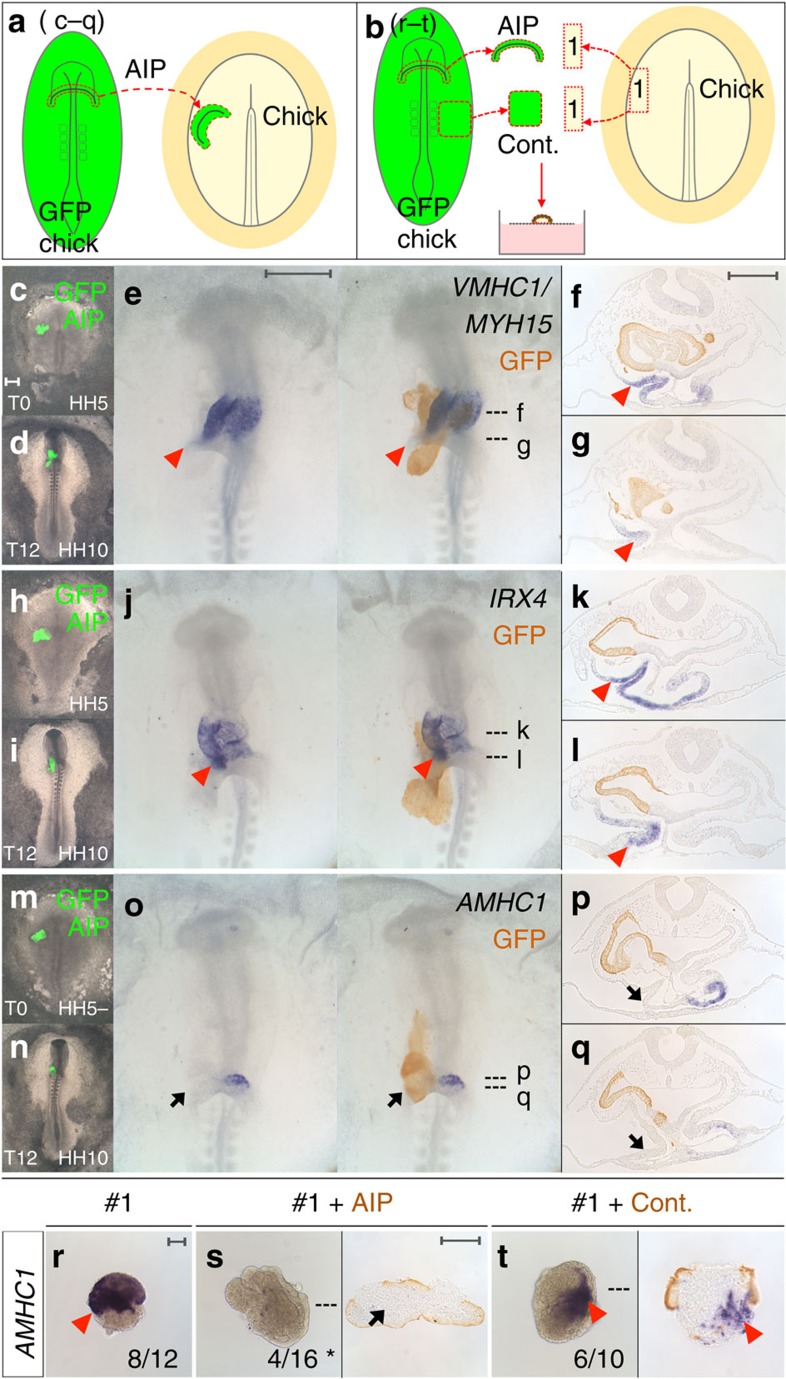
AIP specifies ventricular regional identity and represses atrial character. (**a**) AIP (green, **c**,**d**,**h**,**i**,**m**,**n**; brown, **e**–**g**,**j**–**l**,**o**–**q**) from a HH8 GFP chick was grafted heterotopically onto cardiogenic mesoderm of a HH5 wild-type chick host (**c**,**h**,**m**, T0) and grown for 12 h (**d**,**i**,**n**, T12). (**c**–**q**) AIP expands *VMHC1/MYH15* (**e**–**g**) and *IRX4* (**j**–**l**) expression (red arrowheads) and abolishes *AMHC1* (**o**–**q**, black arrows). (**b**) *In vitro* co-culture of #1-mesoderm from a HH5 chick with HH8 GFP-AIP or control (Cont.) endoderm (brown) for 48 h. *AMHC1* expression in #1-mesoderm (**r**, red arrowhead). AIP represses *AMHC1* in #1-mesoderm (**s**, black arrow), Cont. does not (**t**, red arrowhead). *P≤0.05 using two-tailed Fisher's exact test. Scale bars, 0.5 mm in whole-mounts and 0.1 mm in explants and sections.

To test if the AIP patterns the heart tube by specifying ventricular identity, AIP from transgenic GFP donors was grafted over the cardiogenic mesoderm of a host embryo (Fig. 3a). Strikingly, this expands the expression of *VMHC1*/*MYH15* and *IRX4* posteriorly into the prospective atrial region (Fig. 3c–l), while expression of the atrial marker *AMHC1* is abolished (Fig. 3m–q). Moreover, the AIP represses *AMHC1* in #1-mesoderm which would normally express it (Fig. 3r–t and Supplementary Table 3); since IRX4 is known to repress *AMHC1* (refs 26, 30), this repression by the AIP may be either direct or indirect, due to prior AIP-mediated induction of *IRX4*. Thus, the AIP not only induces ventricular identity, but also actively blocks atrial character. The ability to induce a new fate in responding tissues and to impart a spatial pattern are properties shared by other known organizers^3^. Taken together, these results show that the AIP endoderm is distinct from other endodermal regions, in that it can both induce cardiac identity in mesoderm not destined for this fate and also pattern heart mesoderm by promoting ventricular and suppressing atrial regional character.

### Common signals between organizers

Grafts of either Hensen’s node^8^, notochord or floor-plate^31^ into the anterior limb bud can induce extra digits, suggesting that at least some signals (an obvious one being SHH) may be common to different organizers. We therefore tested whether the heart-inducing or ventricle-specifying functions of the AIP can be mimicked by other organizers. We find that Hensen’s node induces patchy expression of *VMHC1/MYH15* but not *MYOCD* in #3-mesoderm cultured in a host (Supplementary Fig. 10a,b and Supplementary Table 5), as well as in explants (Supplementary Fig. 10c,d and Supplementary Table 5). Hensen’s node has also been shown to induce ectopic *VMHC1*, but not *AMHC1* in the germinal crescent and the lateral plate mesoderm^32^, comparable to the ability of the AIP to induce ventricular markers. Conversely, to test whether the AIP can mimic the neural inducing activity of Hensen’s node, we grafted AIP endoderm into the area opaca^33^ at HH3^+^: this induces the early neural marker *SOX3*, but not the later neural plate marker *SOX2* (Supplementary Fig. 10e,f). Finally, we tested whether the AIP can mimic a ZPA graft into the anterior wing bud: strikingly, the AIP causes duplication of digit-2 (Supplementary Fig. 10h). Therefore, the AIP is partly interchangeable with other organizers at earlier and later stages of development, some of which could be related to its expression of *SHH* (Fig. 1l) or *FGF8* (ref. 34). However it seems that the ability of organizers to substitute for each other is limited.

### Signals from the AIP endoderm

**Figure 4:**
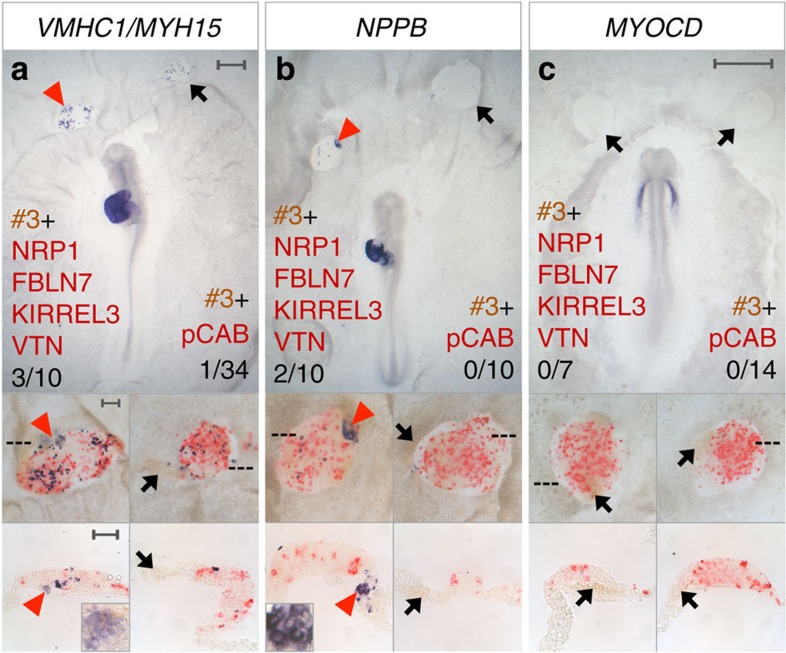
Secreted molecules induce ventricular markers. (**a**–**c**) HH5 quail (brown) #3-mesoderm co-cultured in a chick host with cell pellets transfected with NRP1+FBLN7+KIRREL3+VTN (red) either overnight (**a**,**b**) or for 6–9 h (**c**). *VMHC1/MYH15* and NPPB are induced in #3-mesoderm (**a**,**b**, red arrowheads) but *MYOCD* is not (**c**, black arrows). Control pellets (red; pCAB) do not induce *VMHC1/MYH15*, *NPPB* or *MYOCD* (**a**–**c**, black arrows). Scale bars, 0.5 mm in whole-mounts and 0.1 mm in insets and sections.

The inducing and patterning activities of other known organizers rely on multiple signals, acting together and/or sequentially^35^,^36^,^37^. FGF8 (refs 38, 39) and SHH (refs 40, 41) play important roles in other organizers; both have been implicated in cardiogenesis^34^,^42^ and both are expressed in the AIP. However, our synexpression set contains many other secreted or membrane-associated factors that have not hitherto been implicated either in the activity of other organizers or in cardiac gene induction or heart patterning. Four of these are expressed in the early AIP: *NRP1, FBLN7, KIRREL3* and *VTN* (Fig. 1j,k,m,n). To investigate their activity, we co-cultured #3-mesoderm with pellets of cells transfected with expression plasmids encoding these four factors. This combination partly mimics the AIP, sometimes inducing patchy expression of the ventricular markers *VMHC1/MYH15* (Fig. 4a) and *NPPB* (Fig. 4b), but not *IRX4* (Supplementary Fig. 11f) or other early cardiac markers (Fig. 4c, Supplementary Fig. 11a–e and Supplementary Table 6).

These results suggest that other signals are required, possibly SHH and FGF8, but also considerable complexity, not unlike other organizers, which are believed to emit different signals at different times to account for their various inducing and patterning functions^35^,^36^,^37^. Accordingly, some gene expression profiles change over time as the organizer changes its properties. Although HH8 AIP endoderm was used in co-cultures, the AIP continues its developmental programme, expressing transcripts that are detected in the later AIP (Supplementary Fig. 12). HH8 AIP cultured either alone or with #3-mesoderm expresses genes, including those that encode secreted or membrane-associated molecules normally observed in the HH10 AIP (Supplementary Fig. 4j,k), after 24 h (Supplementary Fig. 12a–d) and those expressed in the HH12-13 AIP (Supplementary Fig. 4q,s) after 48 h (Supplementary Fig. 12e–h). These results suggest that the signalling properties of the AIP endoderm mature over the culture period as they do in the embryo.

Taken together, our results implicate the AIP as an organizer of the heart, and suggest that a complex combination of signals account for its heart inducing and patterning functions at early stages of heart tube formation.

## Discussion

Our results suggest that organizers do share a common genetic signature, and that this property can be used to identify putative new organizers acting during development. The choice of the three organizers to compare was relatively arbitrary and perhaps we should have used a different well-characterized organizer, the mid-hindbrain boundary (MHB) or isthmic organizer^3^,^7^ instead of the comparison between notochord/floor-plate with dorsal neural tube (particularly as the latter is also a signalling region). The main reason why we did not use the MHB is that it is difficult to identify a non-organizer tissue that is similar enough to this boundary in every other respect. The organizer gene set could be refined further using this tissue in future. Nevertheless, this study is an important proof of principle and, based on the synexpression of 48 genes, we uncover the AIP endoderm as a putative organizer because it can both induce cardiac identity and pattern the heart field by specifying regional characteristics (inducing ventricle and suppressing atrial character).

**Figure 5:**
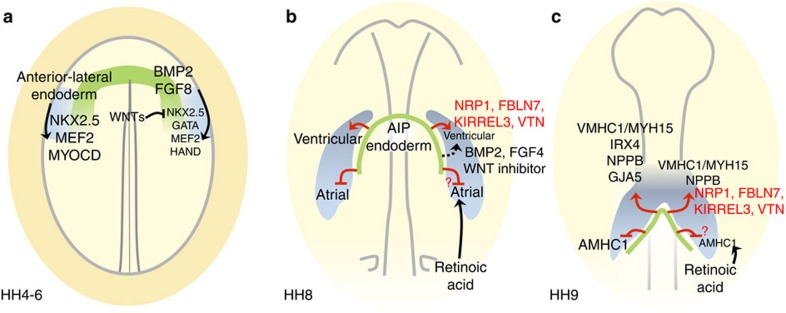
Successive roles of the endoderm in heart induction and patterning. Each diagram represents a stage in development; in each, tissue interactions are depicted on the left, signals on the right. (**a**) Initially (HH 4–6), the anterior-lateral endoderm (including some prospective AIP^56^, green) is required for the adjacent cardiac mesoderm (light blue) to express *NKX2.5*, *MEF2C*^29^ and *MYOCD*^43^. BMP2 and FGF8 signalling from the anterior-lateral endoderm induce *NKX2.5*, *GATA*, *MEF2* and *HAND* transcription factors^22^,^34^,^51^,^52^. The anterior endoderm expresses WNT inhibitors including Crescent, which can induce *NKX2.5* (ref. 49). (**b**) At HH8, progenitor cells are becoming determined as either atrial or ventricular^28^,^57^,^58^. The AIP endoderm induces ventricular character and represses atrial identity. Signals from the AIP endoderm, including NRP1, FBLN7, KIRREL3 and VTN described here (red text and arrows) and previously reported BMP2, FGF4 and Wnt inhibitors^22^,^32^,^49^ (black text and arrows), induce some ventricular markers, which are integrated with atrial-inducing retinoic acid signalling from the posterior lateral plate mesoderm^28^,^57^,^59^. The signals from the AIP endoderm that repress atrial identity are unknown. (**c**) At HH9, when regional markers of anterior–posterior heart tube patterning begin to be expressed, AIP endoderm induces ventricular markers *VMHC1/MYH15*, *IRX4*, *NPPB* and *GJA5* (the latter expressed from HH12) and represses the atrial marker *AMHC1*. Four secreted molecules from the AIP endoderm, NRP1, FBLN7, KIRREL3 and VTN can induce *VMHC1/MYH15* and *NPPB.*

Previous studies have concluded that the endoderm is required for heart development (reviewed in ref. 13). Many of these studies investigated the early anterior-lateral endoderm adjacent to the cardiac mesoderm at HH4-6 (refs 34, 43, 44, 45, 46, 47, 48). Tissue recombination experiments have shown that this endoderm can reprogram non-cardiac tissues to heart identity^29^ and BMPs, FGFs and inhibition of WNT signalling have been implicated in this^13^,^22^,^32^,^34^,^49^,^50^,^51^,^52^,^53^ (Fig. 5a). Some recombination experiments have used posterior primitive streak or posterior lateral plate (#4–5; Supplementary Figs 6a and 7a) explants as a responding tissue to test for cardiac induction^29^,^49^,^50^,^53^. However, it has since been shown that these tissues express *MYOCD* (ref. 43), *GATA* (ref. 54) and *TBX20* (ref. 14) transcription factors, and can express *MYOCD* and *VMHC1/MYH15* following culture *in vitro* (Supplementary Table 1) leaving open the possibility that these experiments do not demonstrate a true change of fate in this tissue. Widespread expression of some early cardiac markers makes it difficult to find a responding tissue suitable for an induction assay (neither being fated to give rise to heart nor express any of the markers being assessed as a result of the induction). Here we use anterior-medial mesoderm (#3), which is neither fated nor specified as heart^20^,^21^,^22^,^23^,^24^ and can be used to assess both induction and patterning rigorously.

The expression patterns of early cardiac markers, like *NKX2.5* (ref. 29), and the signalling molecules previously implicated in their induction (*BMP2/4/7* (ref. 22), *FGF8* (ref. 34) and *CRESCENT* (ref. 49)) indicate that initial signals act at an early stage of cardiogenesis (HH4-6, Fig. 5a) before the AIP forms. Our results show that the AIP from a later stage embryo can also induce heart identity (reprogramming non-heart #3-mesoderm from an earlier stage donor) as well as pattern it by specifying ventricular and suppressing atrial character. During normal development, the AIP is likely to be responsible for the later patterning functions (Fig. 5b), but our results show that it can also mimic the activity of earlier endoderm. This could be due to the presence of AIP precursors within this early endoderm^21^,^55^,^56^ and the continued expression of *BMP2* (ref. 22) and *FGF8* (ref. 34). This is reminiscent of other organizers, for example, Hensen’s node can initiate the entire process of neural induction, even though the first neural inducing signals begin before gastrulation, long before Hensen’s node can be defined^38^. This could be a general feature of organizers, perhaps because they produce many signals. However, the signals emitted by organizers also change over time as their patterning properties evolve, which is seen in organizers like Hensen’s node^11^,^36^, as well as in the AIP.

Heart progenitor cells become determined to either the ventricular or atrial compartment at around HH8 28,57,58(refs 28,57,58), consistent with the timing of signals from the AIP endoderm that induce ventricular and repress atrial character (Fig. 5b). Retinoic acid signalling from the posterior lateral plate mesoderm is known to control the allocation of cardiac progenitor cells to the atrial lineage^28^,^57^,^59^. We have shown that signals from the AIP endoderm, NRP1, FBLN7, KIRREL3 and VTN when combined can sometimes induce *VMHC1/MYH15* and *NPPB* (Fig. 5c), but whether these signals also repress atrial identity remains to be tested. BMP2/4, FGF4 and Wnt inhibitors have also been shown to induce *VMHC1* 22,32,49(refs 22,32,49), although the results of these studies do not rule out induction of heart identity rather than regionalization. Additional regional markers need to be tested to resolve this. Interestingly, both VTN and NRP1 have other, much later roles in heart patterning.

There is conflicting evidence about the extent to which cells that make up the AIP roll around it (involution) and turn over rapidly, or whether they remain in place as it advances caudally down the embryo^19^,^55^,^60^. Neither possibility is incompatible with the AIP being an organizer. The cellular composition of Hensen’s node constantly changes as cells move in and out of it^61^ but it also contains resident cells^62^ and similar findings have been made for the ZPA of the limb^63^. In all these cases the organizer property, as well as molecular markers for it, remain in the region rather than moving with the cells: cells regulate their expression and functional properties according to their current position^61^. These findings raise the general principle that organizers define a position within the embryo where a set of properties come together. Using a ‘synexpression’ gene set for this state should not only allow us to seek new organizers, but also to investigate the clues that instruct cells to have such properties in particular places in the embryo.

Our novel approach of identifying genes common to different organizers offers great potential not only for uncovering new organizing centres during development, but also as a tool to identify new signalling molecules underlying organizer function, as well as transcription factors that confer cells with this property. Interestingly, we find that the synexpression set is enriched for signalling molecules (15 of the 31 enriched genes), whereas transcription factors are more abundant among the depleted genes (6/17), suggesting that some transcriptional repressors may function to suppress the organizer state in non-organizer tissues.

## Methods

### Differential microarray screen and analysis

Fertile Brown Bovan Gold hens' eggs (Henry Stewart, UK) were incubated at 38 °C and staged according to Hamburger and Hamilton (HH)^10^. The following tissues were collected (three separate samples of each) and RNA extracted by standard methods: Hensen’s Node at HH3^+^/4 (HH4 HN) and HH5/6 (HH6 HN), posterior primitive streak at HH3^+^/4 (HH4 PS), HH10/11 notochord and adjacent ventral neural tube/floor-plate (VNT) and the corresponding dorsal neural tube (DNT), the posterior third of the limb at HH20/21 (HH20 PL) and HH24 (HH24 PL) and the anterior third of the limb at HH20/21 (HH20 AL) and HH24 (HH24 AL)^64^.

Hybridization to Affymetrix microarrays was conducted by ARK-Genomics at the Roslin Institute, University of Edinburgh, as described in the Affymetrix GeneChip Expression Analysis Technical Manual (Affymetrix, Inc.). Briefly, 15 μg of total RNA from each sample was reverse-transcribed using a T7-oligo (dT) primer in the first-strand complementary DNA (cDNA) synthesis. After RNAase-H treatment, second-strand cDNA synthesis was carried out. The cDNA was then purified and used as a template in the subsequent *in vitro* transcription reaction for linear amplification of each transcript, and incorporation of biotinylated CTP and UTP using T7 RNA polymerase. The biotinylated cRNA targets were fragmented and hybridized to the Affymetrix GeneChip Chicken Genome Array, which provides good coverage of the chicken genome (32,773 transcripts corresponding to over 28,000 genes). Twenty-seven arrays were used in total (samples from 9 tissues, each in triplicate). Hybridization was performed at 45 **°**C for 16 h with constant rotation (60 rpm). The microarrays were then automatically washed and stained with Streptavidin-phycoerythrin conjugate (SAPE; Invitrogen) in a GeneChip fluidics station (Affymetrix). Fluorescence intensities were measured with a GeneArray scanner 3,000 (Affymetrix). The scanned images were analysed as described in the Affymetrix GeneChip Command Console 3.0 User Manual.

Gene expression data generated from the GeneChip software (GCOS) were normalized using the probe logarithmic intensity error (PLIER) method^65^ within the Affymetrix Expression Console software package. The normalized data were then analysed using the Limma and FARMS^66^ packages within R in Bioconductor. Venn diagrams were generated using the R package Vennerable with dependencies, graph, RBGL, gplots, gtools and reshape. Weights used for scaling the Venn diagrams were based on a priori count data reflecting intersections of genes between different comparisons of datasets.

### Dataset comparisons

Pair-wise comparison of samples was performed as follows:

HH4 HN versus HH4 PS;

HH6 HN versus HH4 PS;

HH4 HN versus HH6 HN;

VNT versus DNT;

HH20 PL versus HH20 AL;

HH24 PL versus HH24 AL.

Probes with a fold change (FC) ≥1.2 (log_2_) and a false discovery rate ≤0.05 were deemed significant. The results of these individual comparisons (using significant enrichment or depletion as the indicator, rather than absolute amounts) were then combined using the Boolean algorithm: ((HH4 HN versus HH4 PS **AND** (HH4 HN versus HH6 HN) **OR** HH6 HN versus HH4 PS)) **AND** VNT versus DNT **AND** (HH20 PL versus HH20 AL **OR** HH24 PL versus HH24 AL). Probes that are differentially enriched or depleted significantly in this algorithm comprise the organizer gene set. The first set of comparisons selects genes enriched or depleted in the early (HH3^+^/4) Hensen’s node compared with the posterior streak, some of which remain expressed the same way at the later (HH5/6) stage. The comparisons for the limb select genes enriched or depleted in the posterior limb bud (containing the ZPA) at either HH20/21 or HH24.

The dataset was submitted to ArrayExpress with the title ‘Microarray analysis of chick embryonic tissues: gastrulation, neural tube/notochord and limb development’ and given accession number E-MTAB-4048.

### Embryo manipulation

Fertilized Brown Bovan Gold hens’ eggs (Henry Stewart, UK), Coturnix coturnix japonica quails’ eggs (B.C. Potter, Rosedean Farm, UK, and Blue Bridge Engineering Limited, Essex, UK) and transgenic GFP chicken eggs^67^ (Roslin Institute, Edinburgh, UK) were used. Embryo manipulations were performed in Pannett-Compton saline and modified New culture^68^,^69^. Tissues were excised from donor embryos using 0.12% w/v trypsin (porcine trypsin 1:250, Sigma) in Pannett-Compton saline and 30 G syringe needles, washed with saline and kept on ice until needed. Mesoderm (#1–5, from both the left and the right sides and used randomly) was harvested from HH5^−^ to HH5^+^ donor embryos. Early AIP endoderm (AIP) and control (Cont.) lateral endoderm (the endoderm underlying the somites and proximal lateral plate mesoderm) were harvested from HH8^−^ to HH8^+^ embryos. Early AIP endoderm was used because cardiac progenitors are closely associated with the AIP at this time, and because it is easier to isolate the AIP at this stage than later, when the heart is beating. Anterior-lateral endoderm (#1-2E) and anterior-medial endoderm (#3E) were harvested from HH5 donor embryos. Host embryos ranged from HH4^+^ to HH6. Grafts were placed on the left or the right side of host embryos; no differences were noted in the results. After grafting, the embryos were incubated for either 6–9 h or overnight (12–18 h). Limb grafts were performed in ovo at HH20-21 and incubated for 5 days^70^.

### Delivery of factors using transfected cells

Chicken *NRP1* (NM_204782.1; F primer CCGCTCTCGGAAGG, R primer CATCCCGATTCTCTG), *FBLN7* (XP_003640934.1, formerly *FIBULIN 7-LIKE*; F primer GGACCATGGCTTCGGGGCTC, R primer CCTGGTCTGCCCTAGAACTCATAGGC), *VTN* (NM_205061.1; F primer CTCTG**G**ATCCTGCTCAGTCACAGTAG with introduced BamHI site, R primer CTGGCGGT**GA**ATT**C**GGGTCTAGC with introduced EcoRI site) and *KIRREL3* (XP_004948018.1; F primer CCTGAGGAATGAGCGCTTTC, R primer GTTCAAACGTGCGTCTGCATC) were cloned by RT-PCR using cDNA synthesized from HH13/14 embryo mRNA. Full-length cDNAs were inserted into the pCAβ-IRES-GFP expression vector.

HEK293T cells were grown in DMEM (Gibco)+10% fetal bovine serum (FBS), 1% GlutaMAX (Gibco) and 1% penicillin/streptomycin (Gibco), and transfected with appropriate expression vectors using polyethylenimine^71^. Pellets of 500 cells were made by recombining cells from individual transfections in a hanging drop culture^72^.

### *In vitro* culture of explants

Tissues were excised from chicken and quail embryos as described above, and cultured *in vitro* for 24 or 48 h at 37 °C with 5% CO_2_ on a 0.4 μm Nucleopore filter (Whatman, WHA110407) floating on DMEM (Gibco) with 10% FBS, 5% chicken embryo extract (US Biological), 1% GlutaMAX (Gibco) and 1% Penicillin/Streptomycin (Gibco)^29^. Tissues were arranged such that the basal surface of the AIP faced the mesoderm explants. Spontaneous beating in 48 h explant co-cultures was recorded with a QImaging Retiga 2000R camera and QCapture Pro software.

### Probes and antibodies

Whole-mount *in situ* hybridization and immunohistochemistry were performed as described^73^,^74^. Probes used were as follows: *MYOCD* (ref. 43), *TBX5* (ref. 75)*, IRX4* (ref. 26), *ISL1* (ref. 76)*, PITX2* (ref. 77), *NXPH1* (ref. 78)*, NRP1* (ref. 79)*, NOTO* (*cNOT1*) (ref. 80), *VTN* (ref. 81), *MOXD1* (*DBHR*) (ref. 82), *CXCL12* (*SDF1*) (ref. 83), *ID2* (ref. 84), *DLX6* (ref. 85) and *SHOX2* (ref 86), kindly provided by the originating laboratories*. AMHC1* and *NKX2.5* were kindly provided by T. Brand and *GATA4* by B. Pain. The cDNA available for *VMHC1 (MYH15)*^27^ is a 2.5 kb cDNA, which also has significant (73%) homology to *MYH7b* (*Slow Myosin 2, SM2*); therefore, a short 0.6 kb probe (*VMHC1/MYH15*, generated after cutting with PstI, where the two sequences differ most) was used instead (see *VMHC1/MYH15* short in Supplementary Fig. 6). Other probes, including *MEF2C* (ChEST776g19), *NPPB* (*ANF*; ChEST509m15) and *GJA5* (*CX40*; ChEST304a4), were transcribed from EST clones (Supplementary Data 1)^87^. Following whole-mount *in situ* hybridization, embryos were incubated in QCPN (Developmental Studies Hybridoma Bank, diluted 1:5) and/or anti-GFP antibody (Life Technologies, A11122, diluted 1:2,000); secondary antibodies used were goat anti-mouse IgG peroxidase (Jackson, 115-035-003), goat anti-rabbit IgG peroxidase (Santa Cruz, sc-2004) or goat anti-rabbit IgG alkaline phosphatase (Upstate, 12–448) diluted 1:1,000. Cryosections of 15 μm or paraffin sections of 10 μm were prepared. For skeletal staining, embryos were stained with Alcian Blue^88^.

### Scoring of co-cultures

Explant cultures (Supplementary Tables 1–6) were considered ‘positive’ if high-level expression was observed throughout the majority of the explant. The *n*-numbers in the figures represent those that were scored as positive. The *n*-numbers that include positive and patchy expression are indicated. Explants with no detectable expression or very weak expression (perhaps due to non-specific staining, constitutive expression and/or marginal induction) were scored as negative. Explants with high-level expression in a small patch (some of which could be due to contamination) were excluded from *P*-value calculations, unless indicated. To calculate *P* values, positive (plus patchy where specified) and negative counts were compared across two conditions (#3 alone versus #3+AIP or #3+Cont.) using two-tailed Fisher's exact test with a 2 × 2 contingency table. *P* values ≤0.05 were deemed significant.

### Data availability

The microarray data have been deposited in the ArrayExpress database under accession code E-MTAB-4048. All other data supporting the findings of this study are available within the article and its Supplementary information files or from the corresponding author upon reasonable request.

## Additional information

**How to cite this article:** Anderson, C. *et al*. A strategy to discover new organizers identifies a putative heart organizer. *Nat. Commun.* 7:12656 doi: 10.1038/ncomms12656 (2016).

## Supplementary Material

Supplementary Information Supplementary Figures 1-12, Supplementary Tables 1-6

Supplementary Movie 1 Spontaneous beating in explants of #3-mesoderm after 48 hours of in vitro co-culture with AIP.

Supplementary Data 1 A microarray screen of organizers compared to non-organizer tissue reveals a putative organizer gene set.
