# Supplementary material for: A strategy to discover new organizers identifies a putative heart organizer
Source: Nat Commun. 2016 Aug 25;7:12656. doi: 10.1038/ncomms12656 (PMC5007377; doi:10.1038/ncomms12656)
Supplement: Supplementary Information — Supplementary Figures 1-12, Supplementary Tables 1-6 [file ncomms12656-s1.pdf]

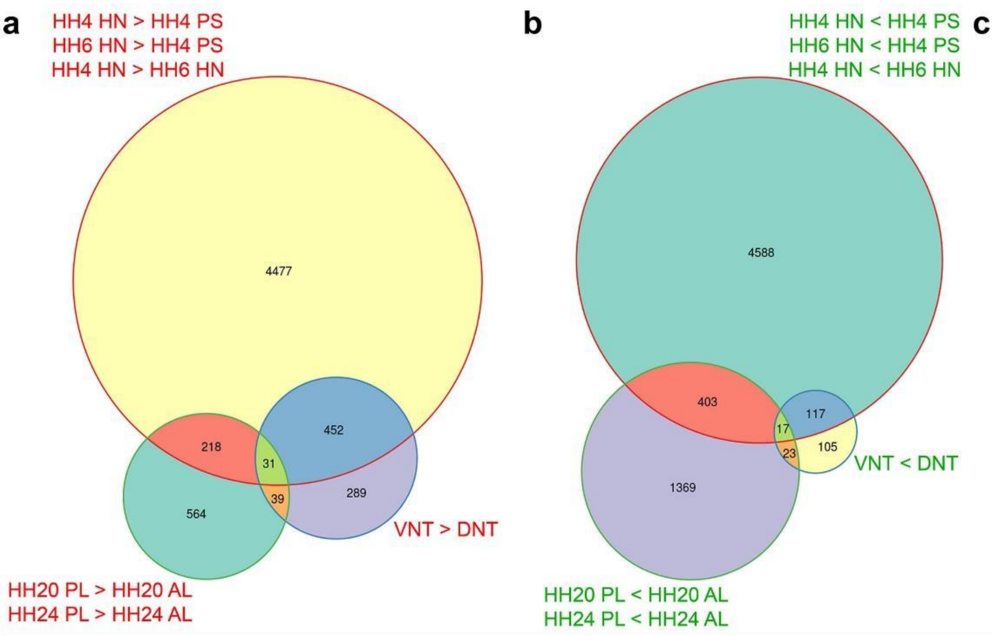

c

Transcription Factor	Intracellular	Membrane/ Secreted
**29 annotated enriched genes**		
NOTO	PKIG	CHGA
LMO7	PPP1R14C	TSPAN6
	GNPDA1	NXPH1
	PIK3R5	PCSK6
	PIK3CD	SHH
	MCF2L	NRSN1
	PLK1S1	TMTC2
	RAB11FIP4	NRP1
	PRKG1	PROM1
		FIBULIN 7
		TMEM63C
		ADRA2A
		VTN
		ENPP4
		SMOC1
		KIRREL3
		SLC39A11
		KCNMA1
7%	31%	62%
+ 2 un-annotated genes		
**16 annotated depleted genes**		
MSX1	MOXD1	PLXNC1
MSX2	SOCS2	CXCL12
DLX5	DSP	CBLN2
DLX6	BTG2	EFNA5
LDB2 (co-factor)	FAM53A	TNFRSF19
ID2		
38%	31%	31%
+ 1 un-annotated gene		

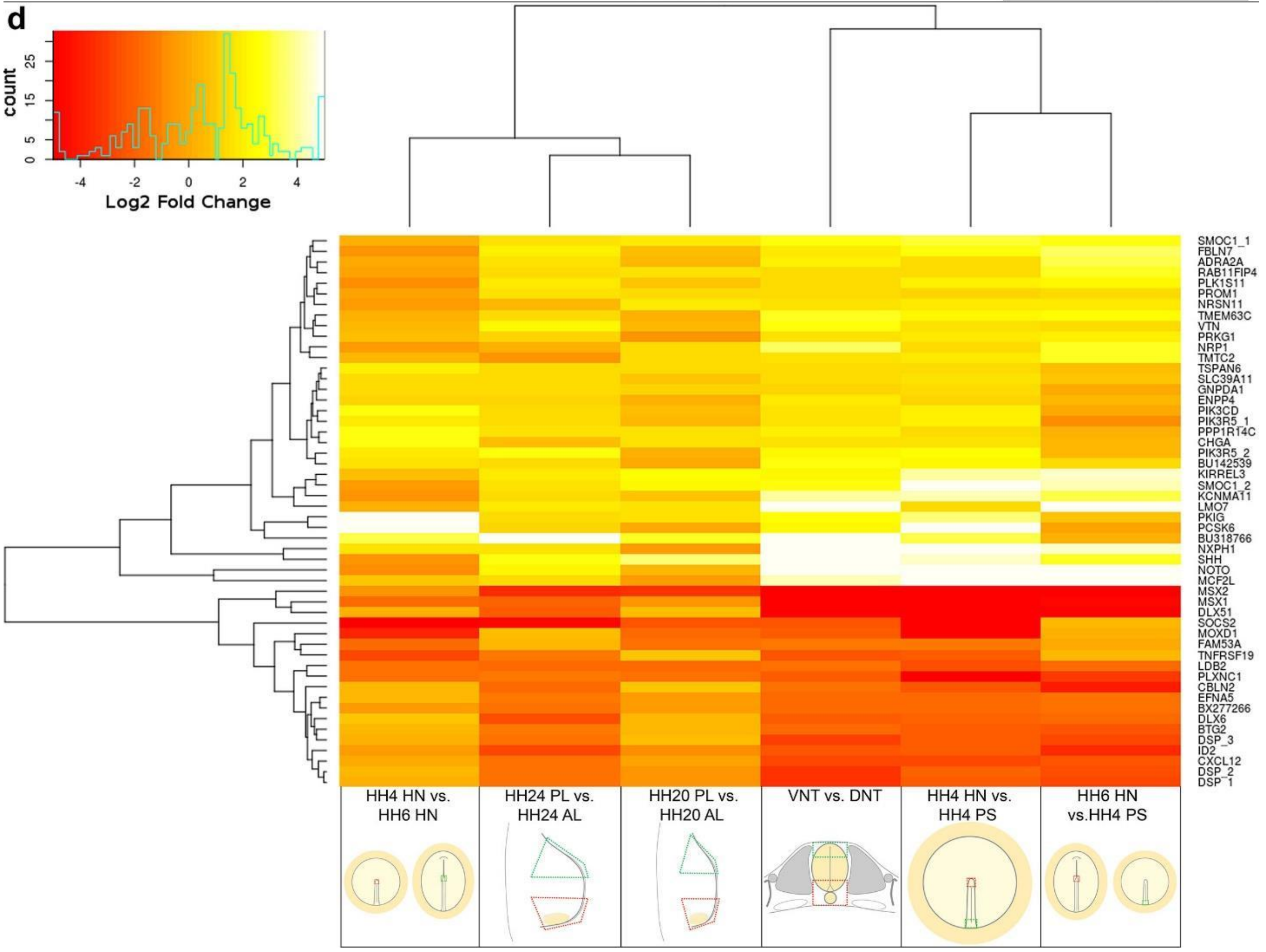

Supplementary Figure 1. A microarray screen of organizers compared to non-organizer tissue reveals a putative organizer gene set. (a, b) Venn diagrams of 31 enriched (a) and 17 depleted (b) genes significantly differentially expressed in organizers compared to their most similar non-organizer tissue. (c) The organizer gene set categorised as transcription factors, intracellular molecules or membrane-associated/secreted molecules. (d) Hierarchical clustering of microarray probes sets. 33 microarray probes enriched in organizers are displayed in white and 19 microarray probes depleted in organizers are displayed in red. The dataset was submitted to ArrayExpress with the accession number E-MTAB-4048. HH4 HN vs. HH4 PS, Hensen's node at HH3^{+}/4 compared to posterior primitive streak at HH3^{+}/4; HH4 HN vs. HH6 HN, Hensen's node at HH3^{+}/4 compared to Hensen's node at HH5/6; HH6 HN vs. HH4 PS, Hensen's node at HH6 compared to posterior primitive streak at HH3^{+}/4; VNT vs. DNT, notochord and ventral neural tube compared to dorsal neural tube at HH10/11; HH20 PL vs. HH20 AL, posterior forelimb compared to anterior forelimb at HH20-1; HH24 PL vs. HH 24 AL, posterior forelimb compared to anterior forelimb at HH24.

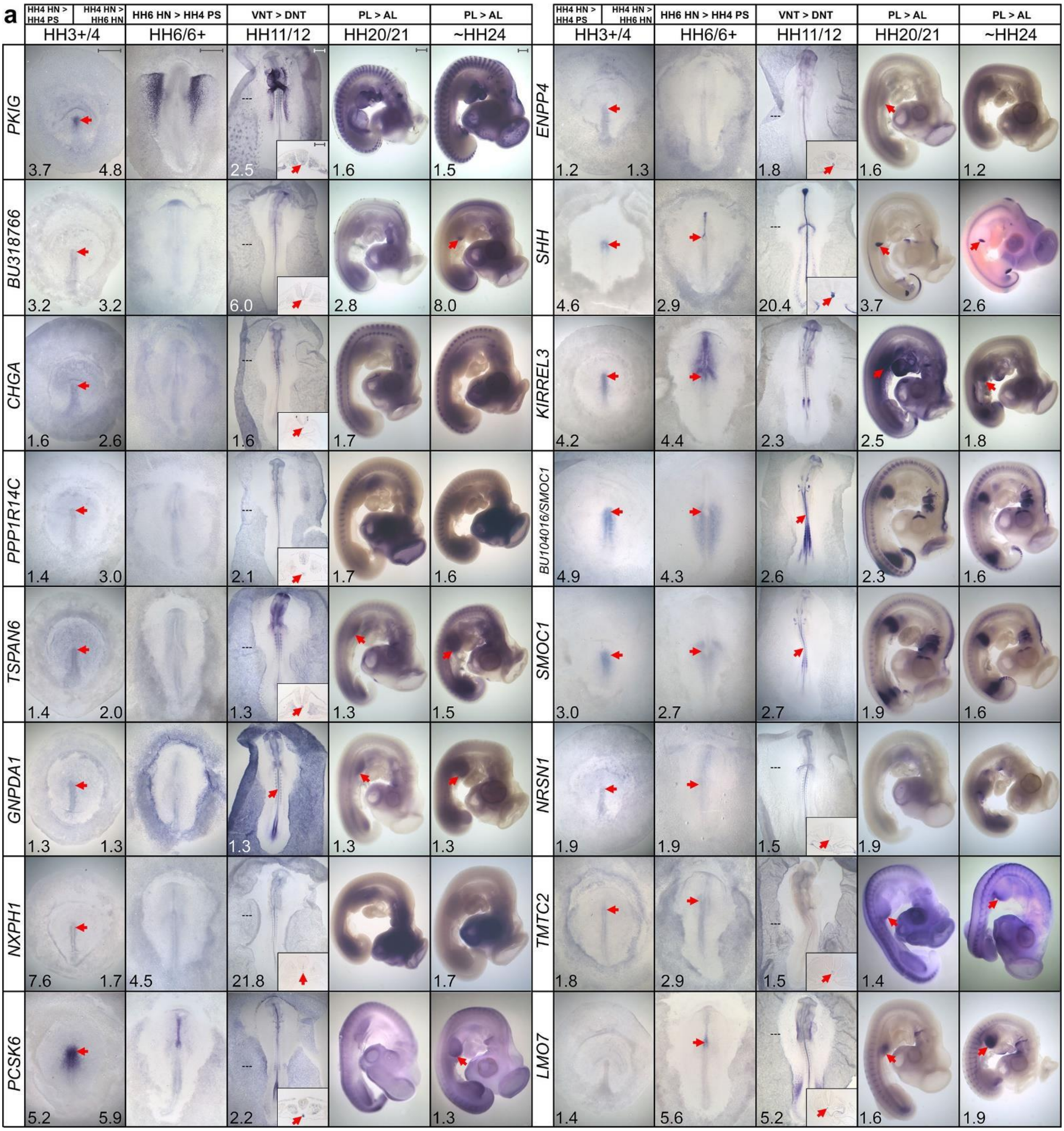
a
HH4 HN > HH4 PS
HH4 HN > HH6 HN
HH6 HN > HH4 PS
VNT > DNT
PL > AL
PL > AL
HH3+/4
HH6/6+
HH11/12
HH20/21
~HH24
PKIG
3.7 4.8 2.5 1.6 1.5
BU318766
3.2 3.2 6.0 2.8 8.0
CHGA
1.6 2.6 1.6 1.7
PPP1R14C
1.4 3.0 2.1 1.7 1.6
TSPAN6
1.4 2.0 1.3 1.3 1.5
GNPDA1
1.3 1.3 1.3 1.3 1.3
NXPH1
7.6 1.7 4.5 21.8 1.7
PCSK6
5.2 5.9 2.2 1.3
ENPP4
1.2 1.3 1.8 1.6 1.2
SHH
4.6 2.9 20.4 3.7 2.6
KIRREL3
4.2 4.4 2.3 2.5 1.8
BU104016/SMOC1
4.9 4.3 2.6 2.3 1.6
SMOC1
3.0 2.7 2.7 1.9 1.6
NRSN1
1.9 1.9 1.5 1.9
TMTC2
1.8 2.9 1.5 1.4
LMO7
1.4 5.6 5.2 1.6 1.9

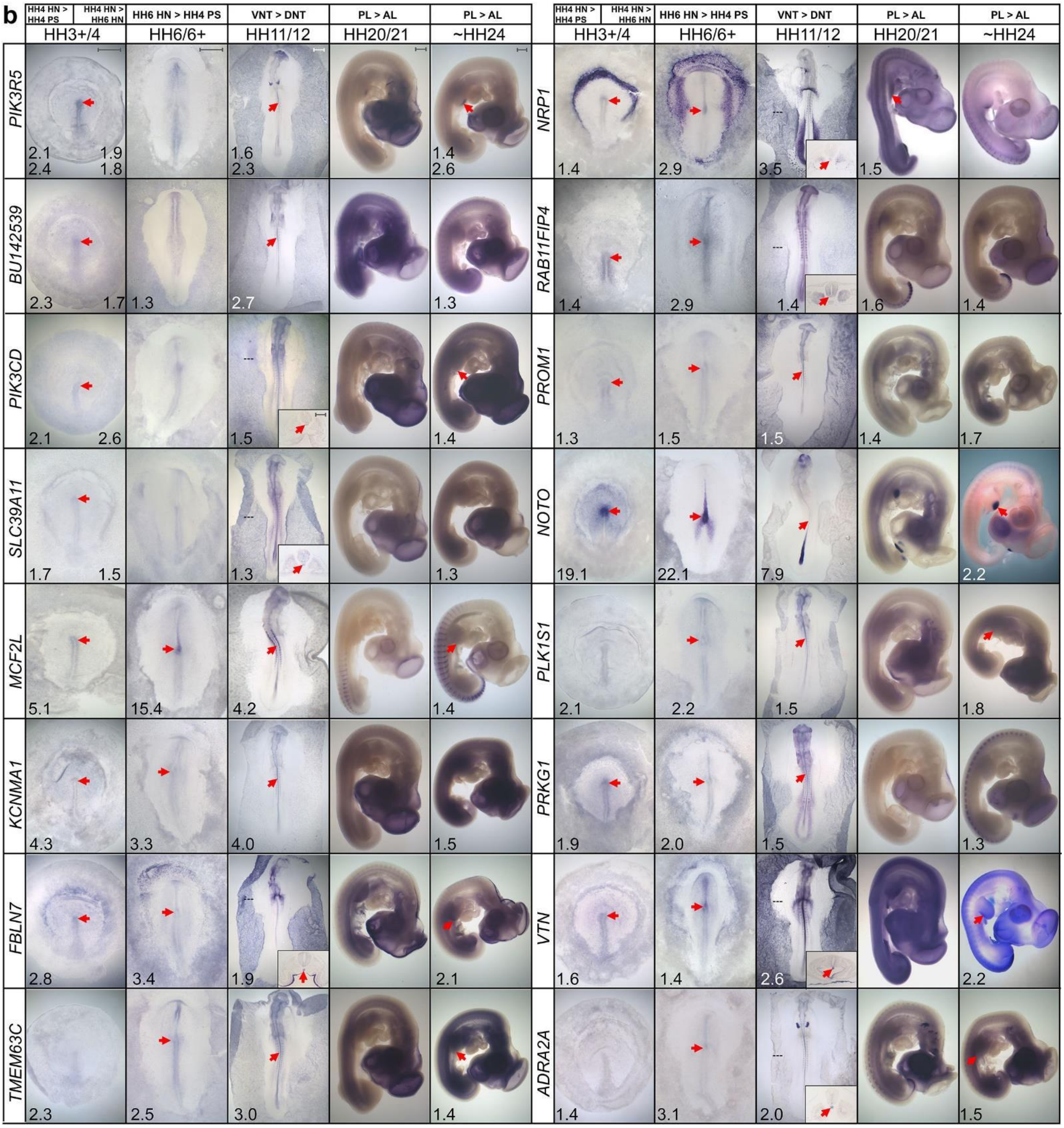

Supplementary Figure 2. In situ hybridisation of enriched transcripts in the putative organizer gene set. (a, b) Verification of the differential microarray screen by mRNA in situ hybridisation of genes enriched in organizers [red arrowheads]. Fold-change values are indicated. Scale bars: 0.5 mm in whole-mounts and 0.1 mm in sections.

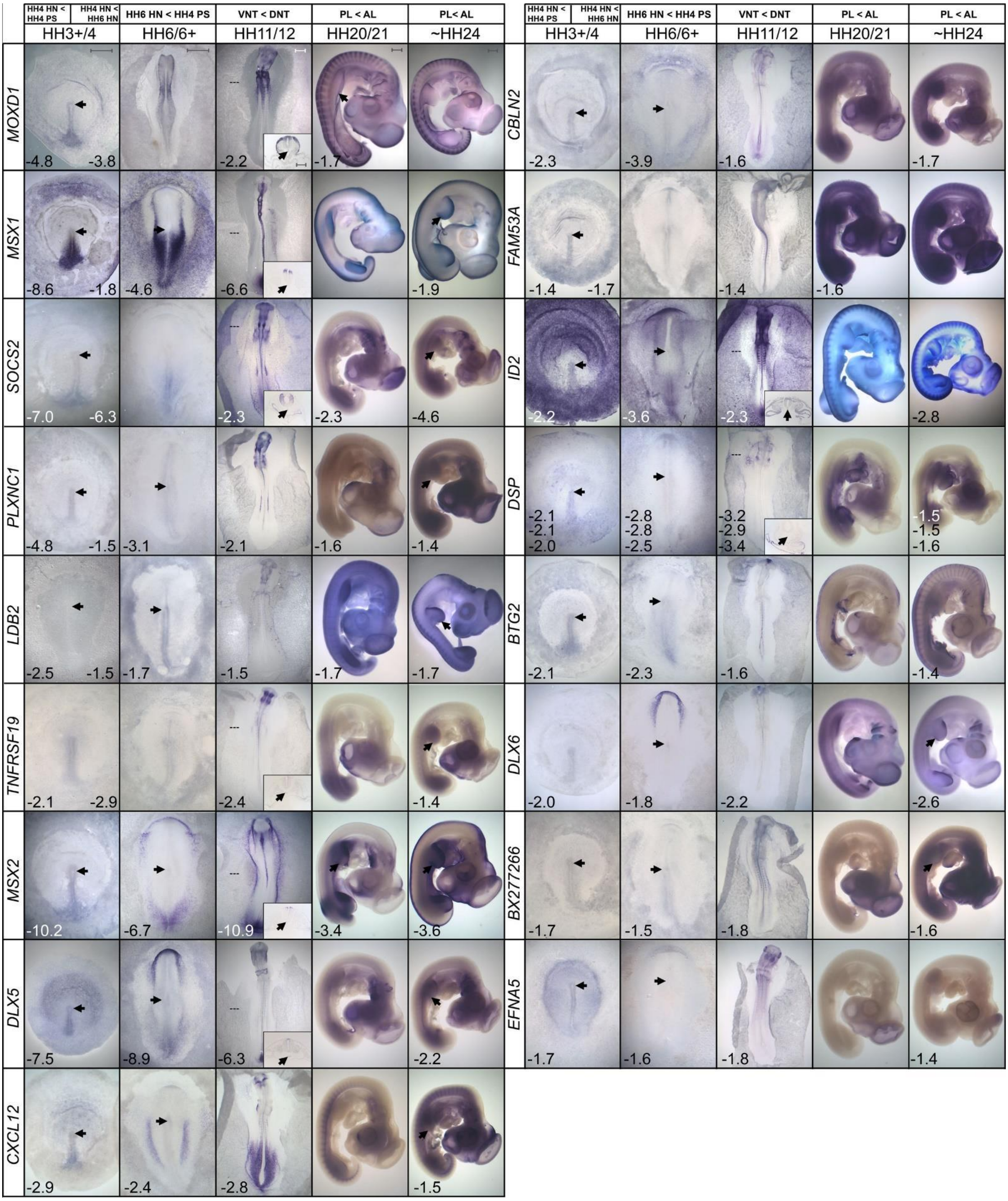

Supplementary Figure 3. In situ hybridisation of depleted transcripts in the putative organizer gene set. Verification of the differential microarray screen by mRNA in situ hybridisation of genes depleted in organizers [black arrows]. Fold-change values are indicated. Scale bars: 0.5 mm in whole-mounts and 0.1 mm in sections.

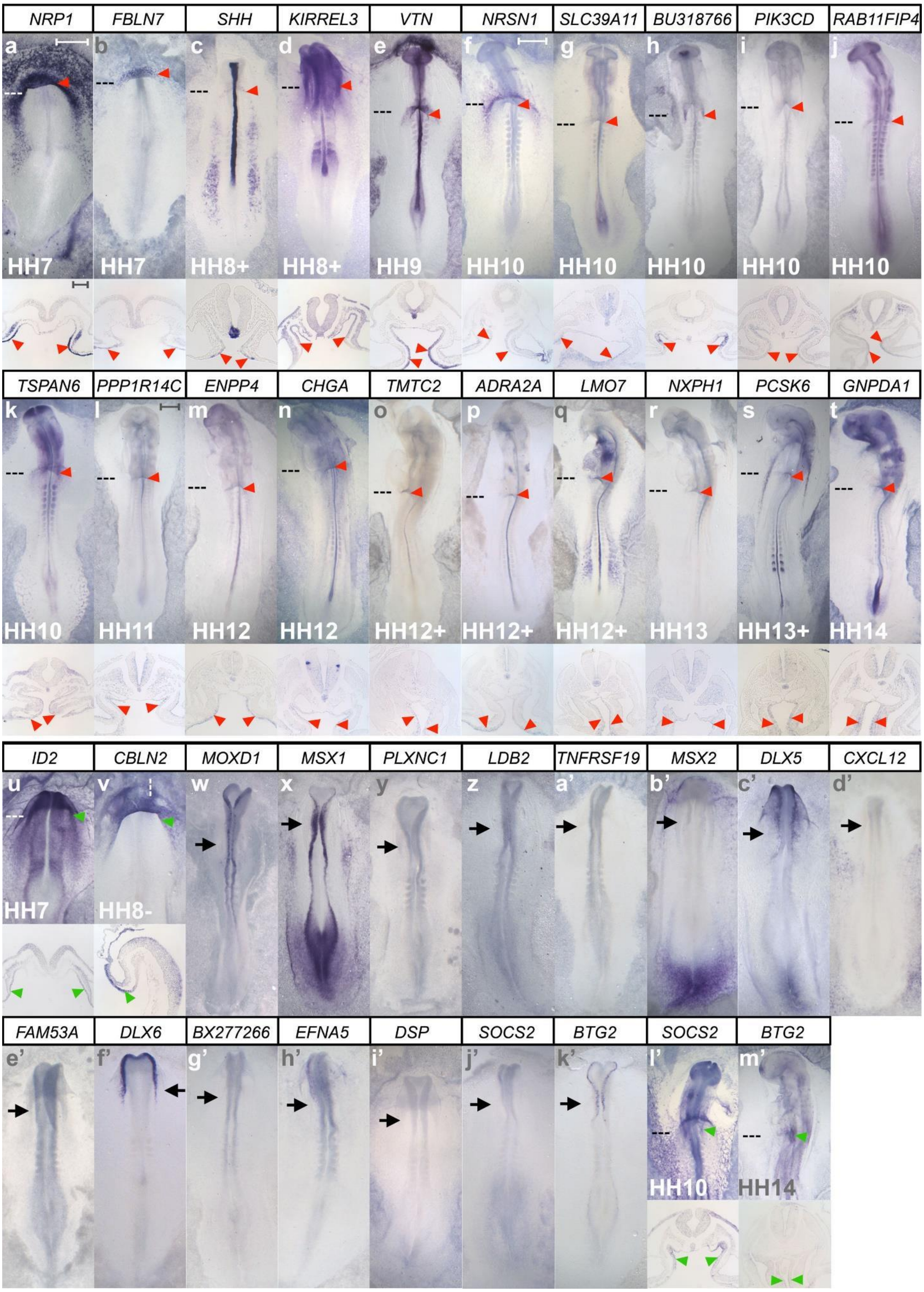

Supplementary Figure 4. The majority of the organizer gene set are appropriately expressed in the AIP. (a-t) 20 transcripts enriched in organizers are expressed in the AIP endoderm [a-t, red arrowheads, ventral]. The stage at which expression is first detected is shown; *NRP1* (a) and *FBLN7* (b) are detected from AIP formation at HH7, *SHH* (c), *KIRREL3* (d) and *VTN* (e) in the early AIP at HH8-9. (u-m') 15 transcripts depleted in organizers are not expressed in the early AIP endoderm [w-k', black arrows, HH8-8+ shown, dorsal]; *ID2* (u, dorsal) and *CBLN2* (v, ventral) are detected from AIP formation [green arrowheads]. *SOCS2* (l') and *BTG2* (m') are detected after HH10 [green arrowheads, ventral]. Scale bars: 0.5 mm in whole-mounts and 0.1 mm in sections.

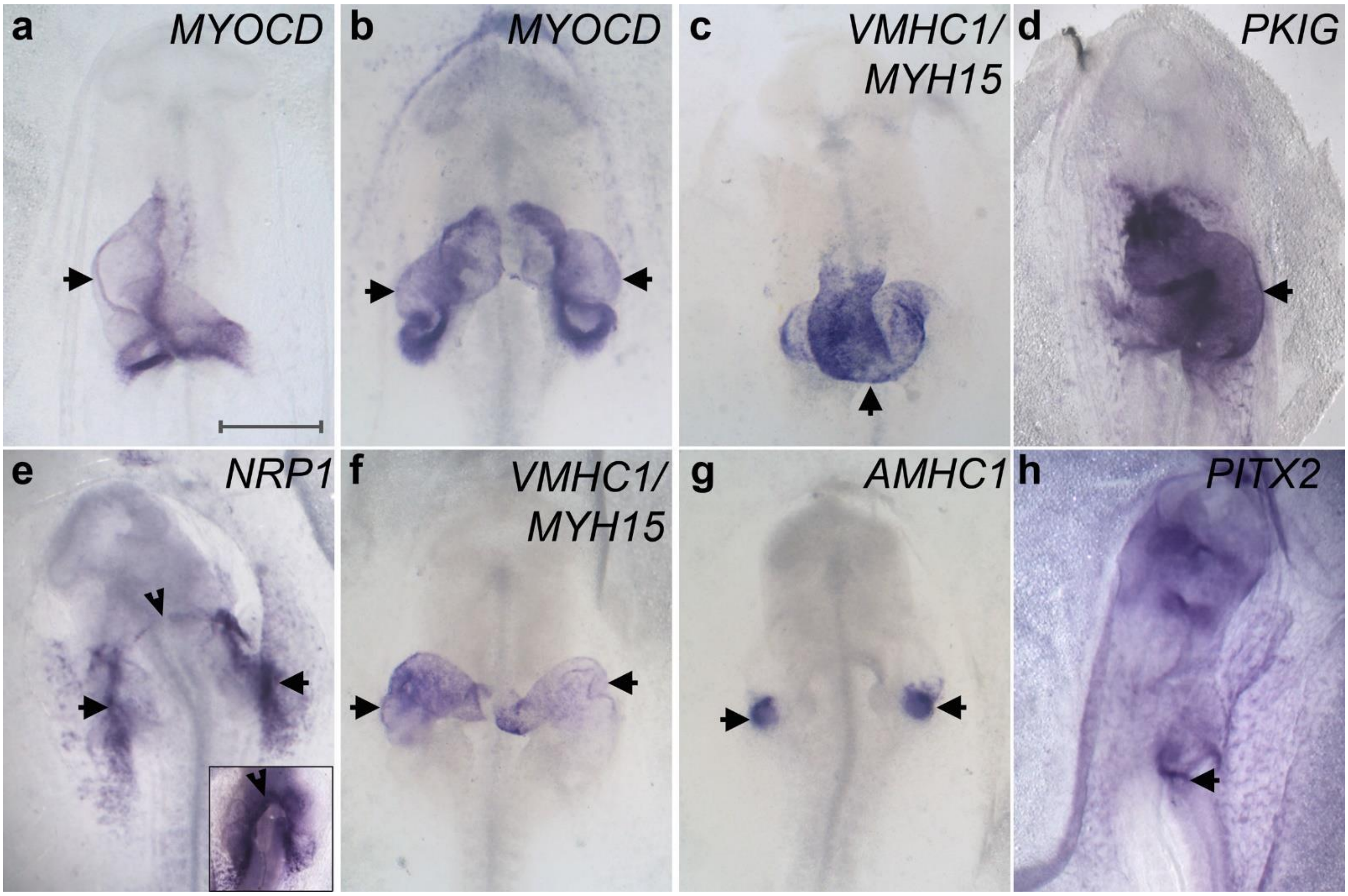

Supplementary Figure 5. Ablation of the AIP at HH8 results in cardia bifida and abnormal heart looping. 48% (24/50) of ablated embryos display cardia bifida [b, e-g, arrows], 20% (10/50) have linear [c, arrow] or reverse-looped hearts [d, h, arrow], 16% (8/50) have normally looped hearts [a, arrow]. 14% (7/50) were indeterminate. *MYOCD* (a, b), *VMHC1/MYH15* (c, f), *AMHC1* (g) and *PKIG* (d) are expressed following AIP ablation at HH8. AIP marker *NRP1* (e) is either normally expressed throughout the healed AIP [arrowhead in inset e] or with a medial gap [e, arrowhead], with no correlation to the observed heart defect. The laterality marker, *PITX2* (g) is correctly expressed in the left LPM [arrow] regardless of abnormal heart looping. Scale bar: 0.5 mm.

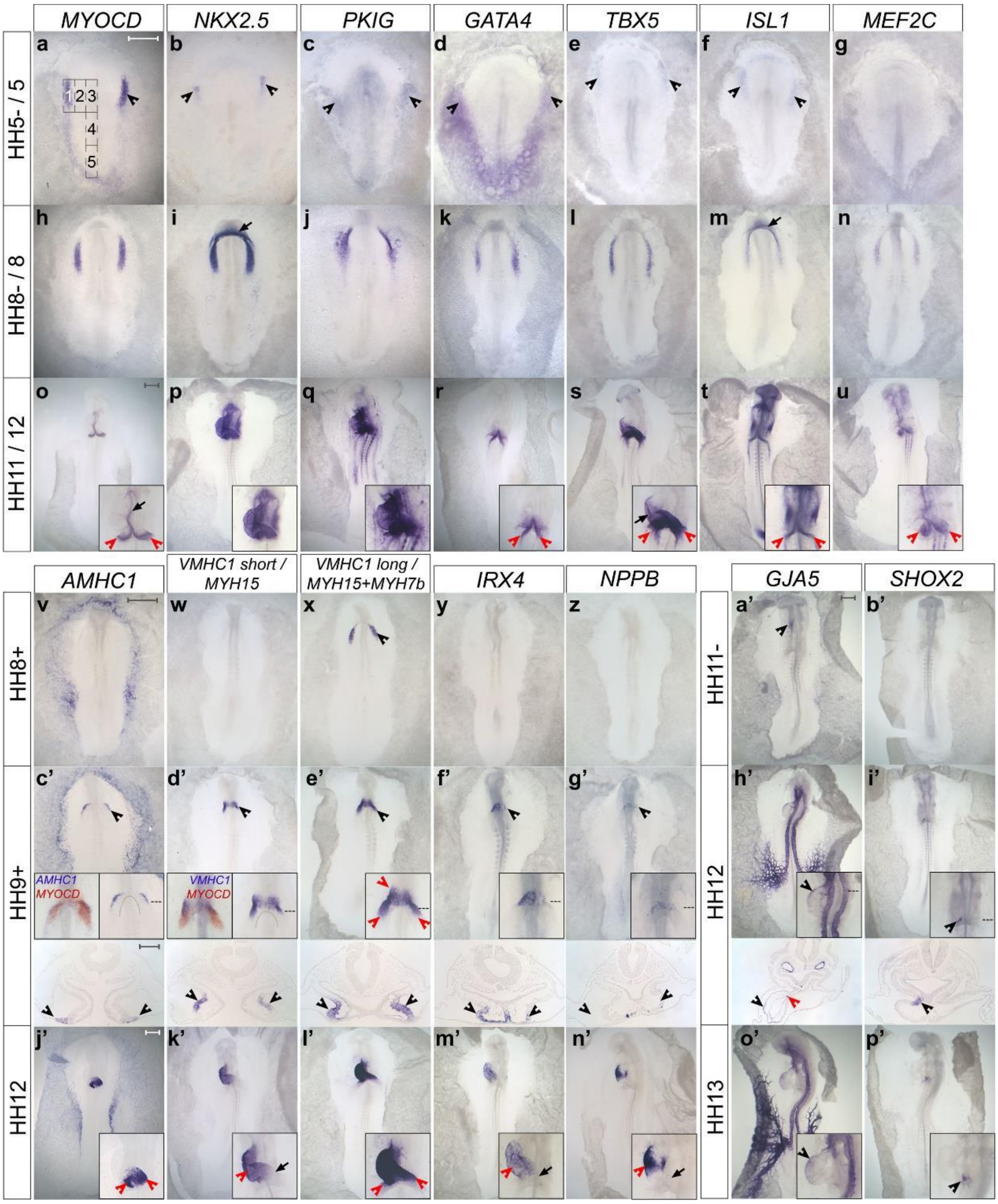

Supplementary Figure 6. Spatio-temporal analysis of cardiac gene expression. (a-u) Early cardiac gene expression. Early cardiac markers, except *MEF2C* (g), are detected bilaterally in the anterior lateral plate mesoderm at HH5-/5 [a-f, arrowheads]. (a) Subdivisions of the HH5 mesoderm; #1 expresses early cardiac markers (a-f); #5 expresses *MYOCD* (a) and *GATA4* (d). (h-n) With AIP formation, cardiac genes are in the splanchnic mesoderm. *NKX2.5* and *ISL1* are detected in the AIP endoderm [i, m, arrows]. (o-u) Expression of early cardiac markers in the developing heart. *NKX2.5* (p) and *PKIG* (q) are throughout the heart tube; *MYOCD* (o), *GATA4* (r) and *ISL1* (t) are in the sinus venosus [red arrowheads]; *TBX5* (s) and *MEF2C* (u) in the prospective atria [red arrowheads]; *TBX5* localises to the prospective left ventricle [s, arrow] and *MYOCD* to the dorsal mesocardium [o, arrow]. (v-n') Expression of regional anterior-posterior heart tube markers begins at HH9 [c', d', f' g', arrowheads]. *AMHC1* (v, c', j') is a marker of the prospective atria [j', red arrowheads]; *VMHC1* short/*MYH15* (w, d', k'), *IRX4* (y, f', m') and *NPPB* (z, g', n') are markers of the prospective ventricles [d', f', g', red arrowheads]. *AMHC1* is first observed in the lateral splanchnic mesoderm [c', arrowhead], *VMHC1* short/*MYH15* is medial [d', arrowhead], *IRX4* and *NPPB* are anterior and medial [f', g', arrowheads]. *VMHC1* long (*MYH15* and *MYH7b;* x, e', l') is observed earlier at HH8+ [x, arrowhead], broadly [e', red arrowheads] and in the prospective atria [l', red arrowheads], compared to *VMHC1* short/*MYH15, IRX4* and *NPPB* which are not in the prospective atria [k', m', n', arrows]. (a', h', o') Expression of *GJA5* begins at HH11- in endothelial cells of the outflow tract [a', arrowhead] and is detected in the endocardium [h', red arrowhead] and anterior myocardium [h', o', black arrowheads]. (b', i', p') *SHOX2* is a marker of the sinoatrial node and is detected from HH12 in the sinus venous [i', black arrowheads]. Scale bars: 0.5 mm in whole-mounts and 0.1 mm in sections.

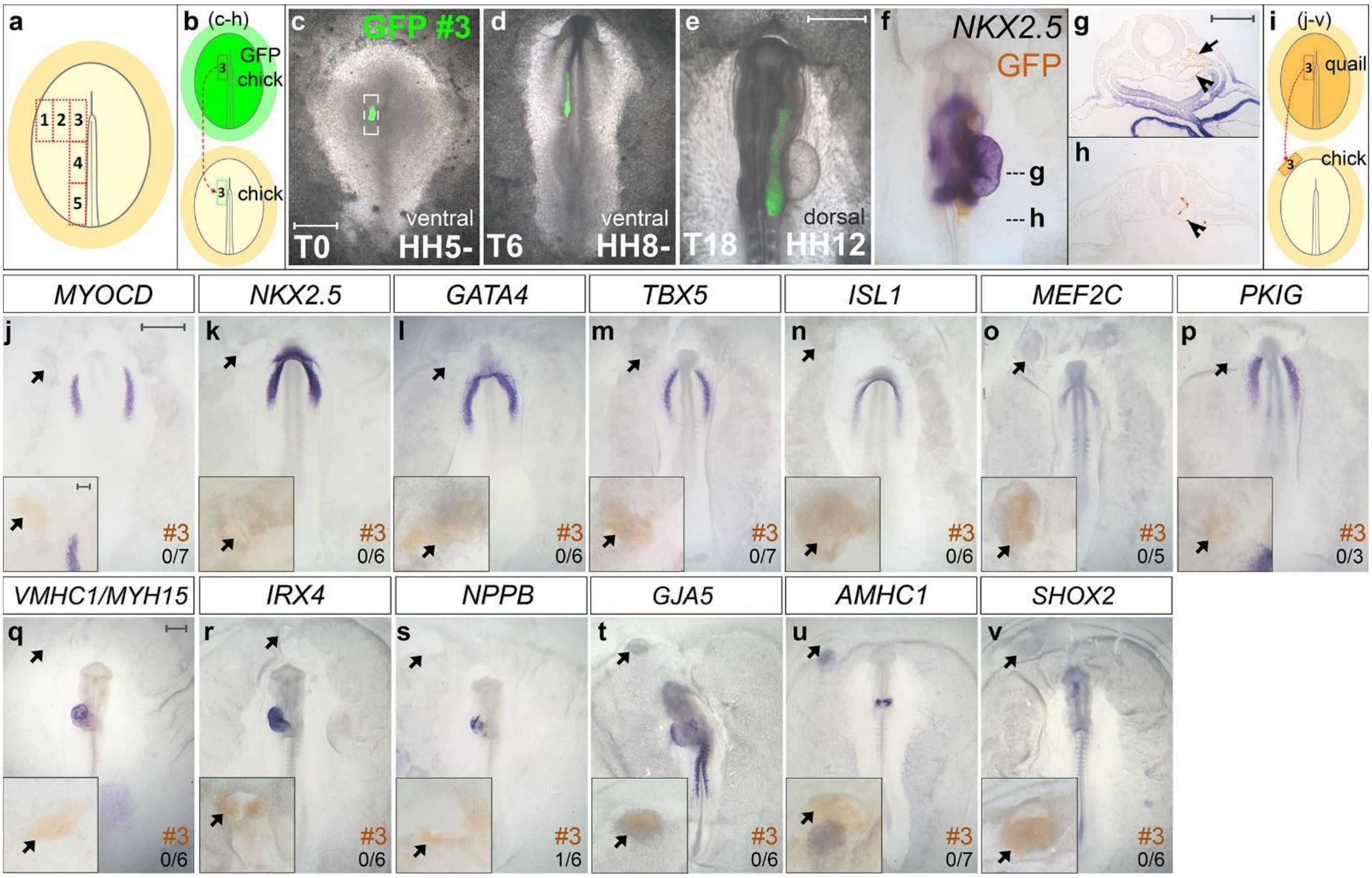

Supplementary Figure 7. Anterior-medial mesoderm is not fated or specified as heart. (a) Subdivisions of the HH5 mesoderm. (b) Anterior-medial mesoderm (#3) from a HH5 GFP chick grafted homotopically into a wild-type chick host (c-h). GFP #3-mesoderm [c-e, green; f-h brown] in a chick host [c, T0] after 6 [d, T6] and 18 hours [e, T18] forms paraxial mesoderm of the head [g, arrow] and endothelial cells of the dorsal aorta [g, h, arrowheads]. (i) Mesoderm-#3 from a HH5 quail embryo cultured in the anterior area opaca of a host chick (j-p: 6-9 hours; q-v: overnight). (j-v) Quail #3-mesoderm (brown) does not express cardiac genes [black arrows]. Scale bars: 0.5 mm in whole-mounts and 0.1 mm in insets and sections.

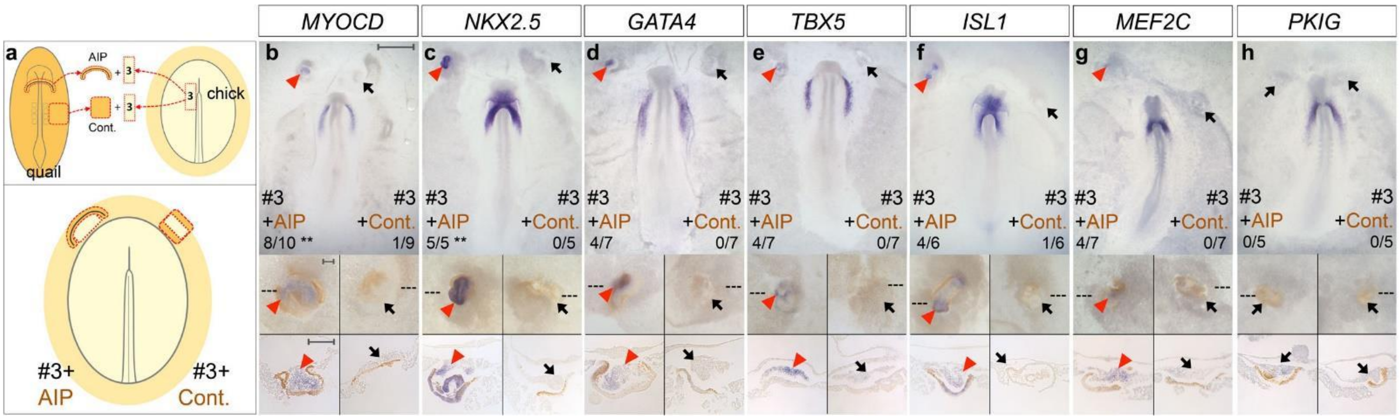

Supplementary Figure 8. AIP induces early cardiac markers in non-cardiac mesoderm. (a) AIP or control (Cont.), lateral endoderm from a HH8 quail co-cultured with chick #3-mesoderm in a host embryo for 6-9 hours. (b-g) Cardiac markers *MYOCD* (b), *NKX2.5* (c), *GATA4* (d), *TBX5* (e), *ISL1* (f), and *MEF2C* (g) are induced in #3-mesoderm [red arrowheads] by quail (brown) AIP but not by control (Cont.), non-AIP endoderm [black arrows]. *PKIG* (h) is not induced [black arrows]. ** $p \leq 0.005$ using two-tailed Fisher's exact test. Scale bars: 0.5 mm in whole-mounts and 0.1 mm in insets and sections.

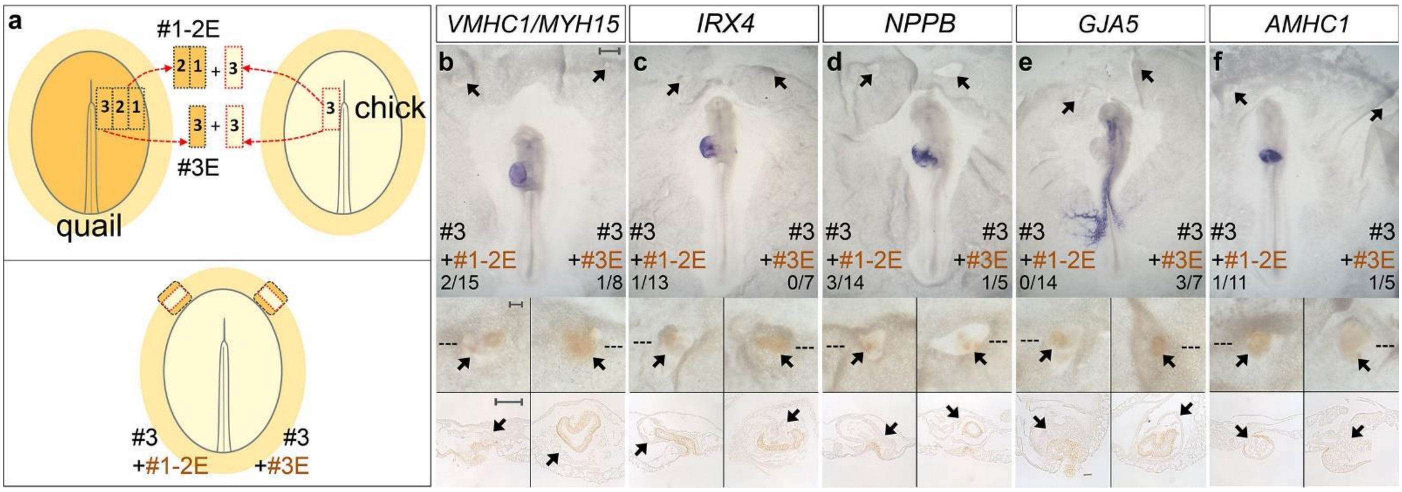

Supplementary Figure 9. Anterior-lateral endoderm does not induce regional cardiac markers in non-cardiac mesoderm. (a) Anterior-lateral endoderm (#1-2E) or control, anterior-medial endoderm (#3E) from a HH5 quail embryo co-cultured with chick #3-mesoderm in a host embryo overnight. (b-f) Regional heart tube markers *VMHC1/MYH15* (b), *IRX4* (c), *NPPB* (d), *GJA5* (e) and *AMHC1* (f) are not induced in #3-mesoderm by quail (brown) #1-2E or by #3E [black arrows]. Scale bars: 0.5 mm in whole-mounts and 0.1 mm in inserts and sections.

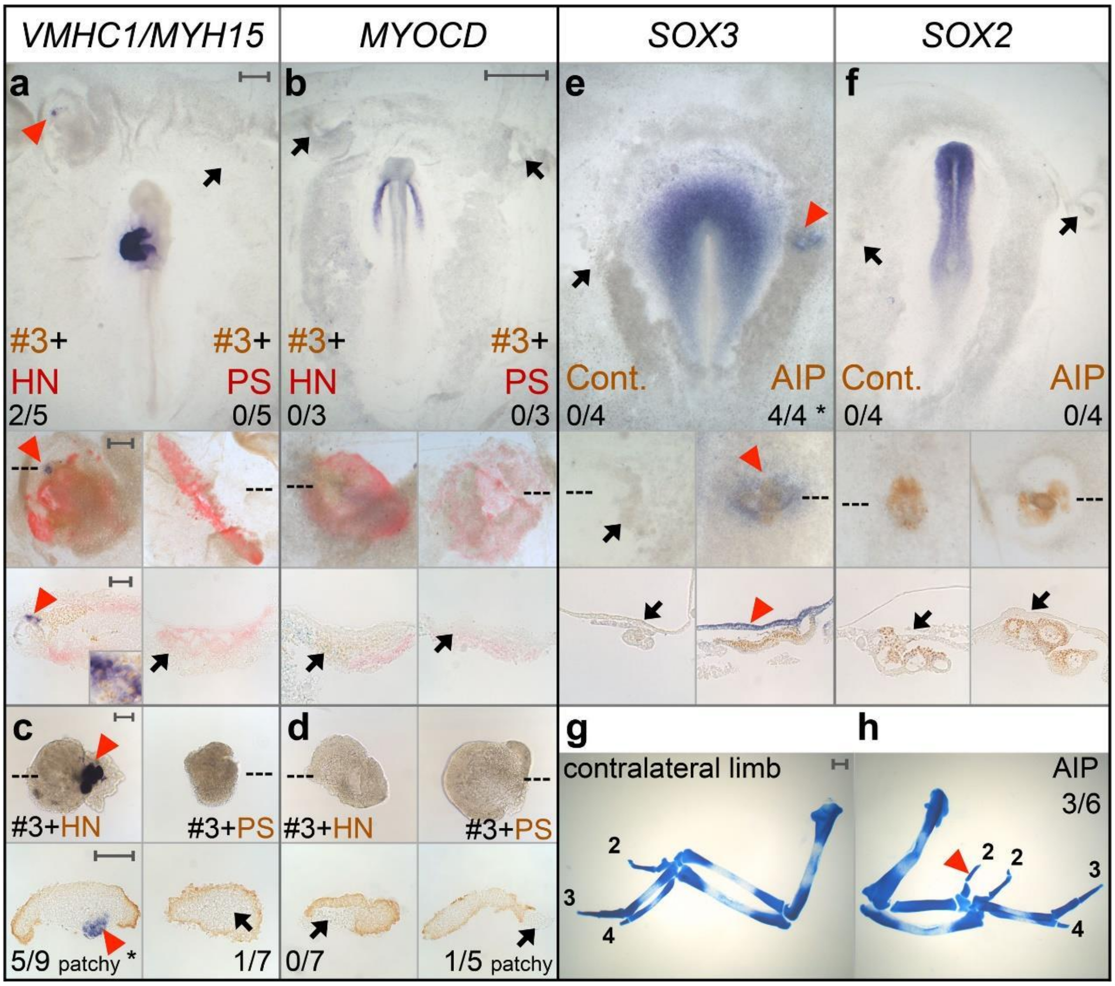

Supplementary Figure 10. AIP is partly interchangeable with other organizers. Hensen's node (HN) induces *VMHC1/MYH15* [a, c, red arrowheads] but not *MYOCD* [b, d, black arrows] in #3-mesoderm co-cultures either in a host chick embryo (a: overnight; b: 6-9 hours) or in vitro (c: 48 hours; d: 24 hours); control posterior streak (PS) induces neither [a-d, black arrows]. AIP induces *SOX3* [e, red arrowheads] but not *SOX2* [f, black arrows] in epiblast. Control (Cont.), non-AIP endoderm induces neither [e, f; black arrows]. (g, h) AIP duplicates digit 2 in the wing [red arrowhead]. * $p \leq 0.05$ using two-tailed Fisher's exact test. Scale bars: 0.5 mm in whole-mounts and 0.1 mm in inserts, explants and sections.

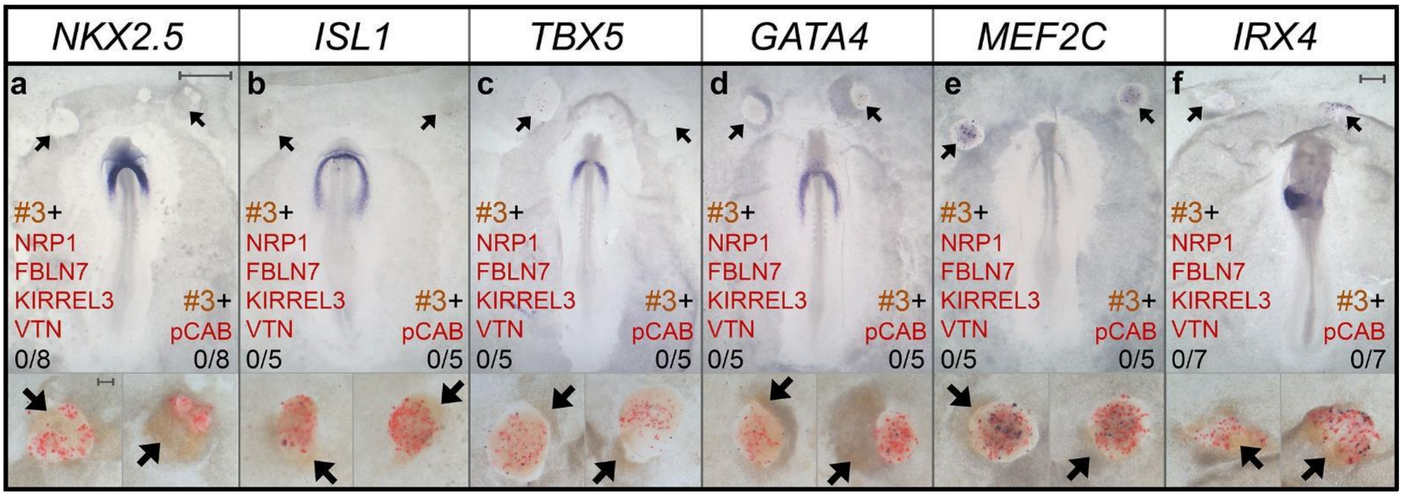

Supplementary Figure 11. Secreted molecules do not induce early cardiac markers. (a-f) HH5 quail (brown) #3-mesoderm co-cultured in a chick host with cell pellets transfected with NRP1+FBLN7+KIRREL3+VTN (red) for 6-9 hours (a-e) or overnight (f). Cell pellets releasing NRP1+FBLN7+KIRREL3+VTN and control pellets (pCAB) do not induce *NKX2.5* (a), *GATA4* (b), *TBX5* (c), *ISL1* (d), *MEF2C* (e) or *IRX4* (f) in #3-mesoderm [a-f, black arrows]. Scale bars: 0.5 mm in whole-mounts and 0.1 mm in inserts.

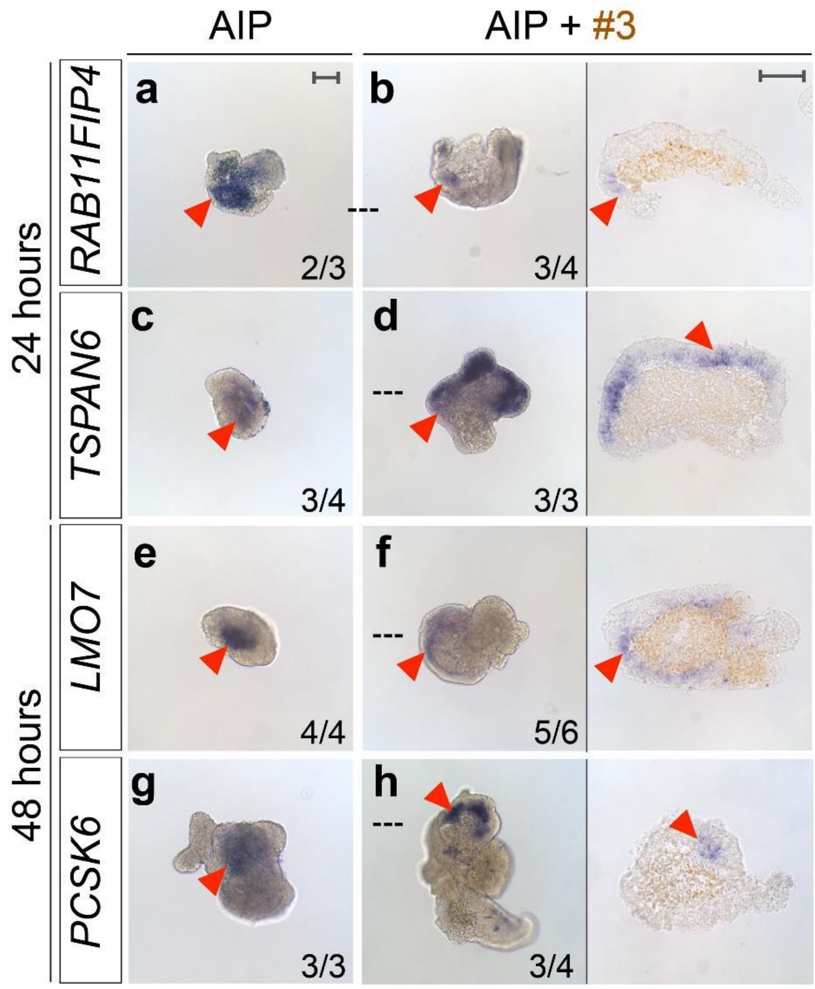

Supplementary Figure 12. AIP character matures in vitro. In vitro culture of HH8 chick AIP alone (a, c, e, g) or co-cultured with HH5 quail #3-mesoderm [brown, b, d, f, h] for 24 (a-d) or 48 hours (e-h). Transcripts normally detected in HH10 AIP, *RAB11FIP4* (a, b) and *TSPAN6* (c, d) are detected in AIP after 24 hours of culture [red arrowheads]. Transcripts normally detected in AIP from HH12+, *LMO7* (e, f) and *PCSK6* (g, h) are detected in AIP after 48 hours [red arrowheads]. Scale bars: 0.1 mm.

Supplementary Table 1. Mesoderm #3 does not express cardiac genes when cultured in vitro.

	positive	patchy	weak	negative	total	% positive
			MYOCD			
**#1** 24 hour	8	0	2	2	12	67%
**#2** 24 hour	6	1	1	3	11	55%
**#3** 24 hour	1	0	1	13	15	7%
**#4** 24 hour	6	0	1	8	15	40%
**#5** 24 hour	2	0	2	4	8	25%
**#1** 48 hour	0	0	5	8	13	0%
**#2** 48 hour	1	0	5	8	14	7%
**#3** 48 hour	0	3	1	11	15	0%
**#4** 48 hour	3	0	0	3	6	50%
**#5** 48 hour	2	1	0	2	5	40%
			NKX2.5			
**#1** 24 hour	3	0	2	0	5	60%
**#2** 24 hour	0	0	5	2	7	0%
**#3** 24 hour	0	0	0	8	8	0%
**#1** 48 hour	8	0	1	0	9	89%
**#2** 48 hour	6	0	2	0	8	75%
**#3** 48 hour	0	0	1	5	6	0%
			VMHC1/MYH15			
**#1** 24 hour	0	0	0	10	10	0%
**#2** 24 hour	3	0	0	4	7	43%
**#3** 24 hour	0	0	0	7	7	0%
**#4** 24 hour	2	0	0	7	9	22%
**#5** 24 hour	0	0	0	7	7	0%
**#1** 48 hour	7	0	0	0	7	100%
**#2** 48 hour	5	0	0	0	5	100%
**#3** 48 hour	0	0	0	10	10	0%
**#4** 48 hour	2	0	1	4	7	29%
**#5** 48 hour	0	0	1	7	8	0%
			IRX4			
**#1** 24 hour	0	0	0	4	4	0%
**#2** 24 hour	3	1	0	2	6	50%
**#3** 24 hour	0	0	0	8	8	0%
**#1** 48 hour	7	0	2	3	12	58%
**#2** 48 hour	1	0	4	5	10	10%
**#3** 48 hour	0	0	0	7	7	0%
**#4** 48 hour	0	0	0	8	8	0%
**#5** 48 hour	0	0	0	7	7	0%
			NPPB			
**#1** 48 hour	6	0	0	0	6	100%
**#2** 48 hour	0	6	0	0	6	0%
**#3** 48 hour	0	0	0	8	8	0%
			GJA5			
**#1** 48 hour	4	3	0	0	7	57%
**#2** 48 hour	2	4	0	1	7	29%
**#3** 48 hour	0	0	0	7	7	0%
			AMHC1			
**#1** 24 hour	4	1	2	3	10	40%
**#2** 24 hour	3	0	1	3	7	43%
**#3** 24 hour	0	0	0	14	14	0%
**#1** 48 hour	8	2	1	1	12	67%
**#2** 48 hour	1	0	3	2	6	17%
**#3** 48 hour	0	0	1	7	8	0%
			SHOX2			
**#1** 48 hour	0	0	0	6	6	0%
**#2** 48 hour	0	1	0	4	5	0%
**#3** 48 hour	0	0	1	6	7	0%

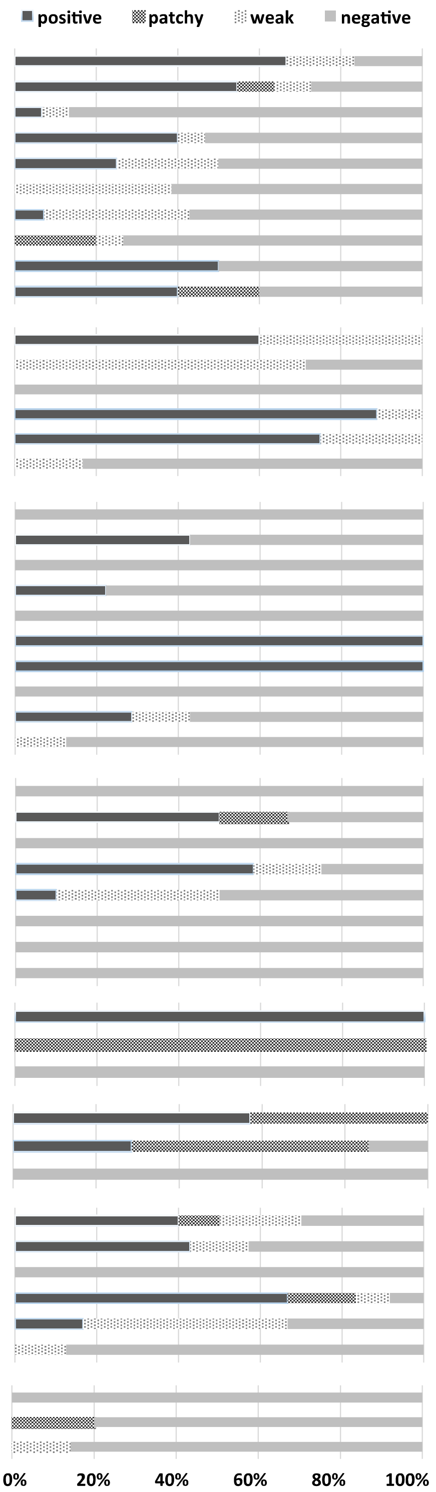

Mesoderm explants #1-5 cultured in vitro for 24 and 48 hours and scored for the expression of cardiac markers. Explants of #3-mesoderm are not scored as positive for expression of *NKX2.5, VMHC/MYH15, IRX4, NPPB*, *GJA5*, *AMHC1* or *SHOX2*. 7% of explants are scored as positive for expression of *MYOCD*. Percentage stacked histograms are depicted for each.

Supplementary Table 2. AIP induces cardiac markers in non-cardiac mesoderm explants.

	positive	patchy	weak	negative	total	p-value	% positive
				MYOCD			
#3	0	0	0	7	7		0%
#3+AIP	8	0	1	1	10	**0.002**	80%
#3+Cont.	1	0	0	8	9	1.000	11%
				NKX2.5			
#3	0	0	0	6	6		0%
#3+AIP	5	0	0	0	5	**0.002**	100%
#3+Cont.	0	0	0	5	5	1.000	0%
				GATA4			
#3	0	0	0	6	6		0%
#3+AIP	4	0	1	2	7	0.070	57%
#3+Cont.	0	0	0	7	7	1.000	0%
				TBX5			
#3	0	0	0	7	7		0%
#3+AIP	4	0	2	1	7	0.070	57%
#3+Cont.	0	0	1	6	7	1.000	0%
				ISL1			
#3	0	0	0	6	6		0%
#3+AIP	4	0	0	2	6	0.061	67%
#3+Cont.	1	0	1	4	6	1.000	17%
				MEF2C			
#3	0	0	0	5	5		0%
#3+AIP	4	0	1	2	7	0.081	57%
#3+Cont.	0	0	2	5	7	1.000	0%
				PKIG			
#3	0	0	0	3	3		0%
#3+AIP	0	0	0	5	5	1.000	0%
#3+Cont.	0	0	0	5	5	1.000	0%
				VMHC1/MYH15			
#3	0	0	0	6	6		0%
#3+AIP	6	0	0	2	8	**0.010**	75%
#3+Cont.	0	0	0	8	8	1.000	0%
				IRX4			
#3	0	0	0	6	6		0%
#3+AIP	4	1	1	0	6	**0.015**	67%
#3+Cont.	0	0	4	2	6	1.000	0%
				NPPB			
#3	1	0	1	4	6		17%
#3+AIP	7	2	0	2	11	**0.041**	64%
#3+Cont.	0	1	1	9	11	0.375	0%
				GJA5			
#3	0	0	0	6	6		0%
#3+AIP	4	4	0	1	9	**0.015**	44%
#3+Cont.	0	3	1	5	9	1.000	0%
				AMHC1			
#3	0	0	0	7	7		0%
#3+AIP	1	1	0	7	9	1.000	11%
#3+Cont.	0	0	0	9	9	1.000	0%
				SHOX2			
#3	0	0	0	6	6		0%
#3+AIP	0	1	0	7	8	1.000	0%
#3+Cont.	2	0	0	6	8	0.473	25%

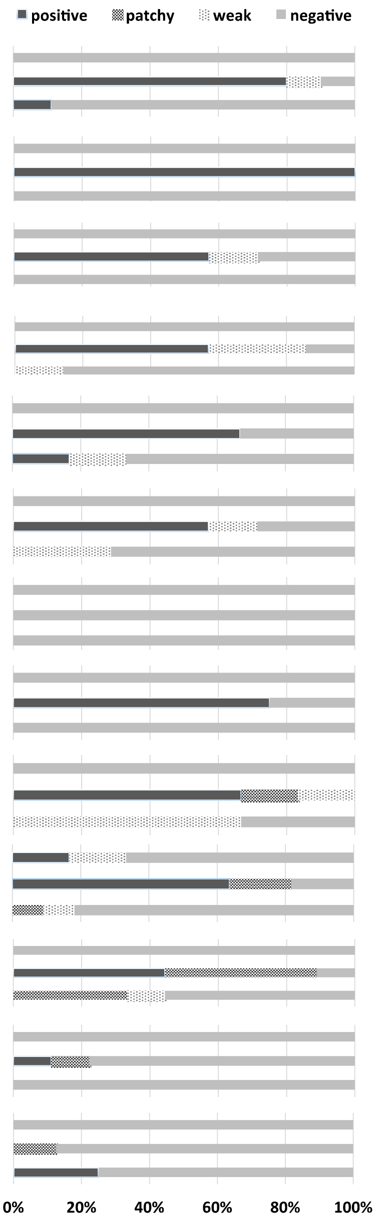

Cardiac markers are not expressed in explants of #3-mesoderm cultured in a host embryo, except 17% of explants are scored as positive for expression of *NPPB*. *MYOCD, NKX2.5*, *VMHC/MYH15, IRX4, NPPB* and *GJA5* are induced in a significant number of #3-mesoderm explants when co-cultured with AIP in a host chick embryo. AIP does not induce *PKIG, AMHC1* or *SHOX2* in #3-mesoderm. Cardiac markers are not induced in #3-mesoderm when co-cultured with control (Cont.), non-AIP endoderm. Percentage stacked histograms are depicted for each condition.

Supplementary Table 3. AIP induces early and ventricular cardiac genes in non-cardiac mesoderm and represses *AMHC1* in cardiac mesoderm.

	positive	patchy	weak	negative	total	p-value	% positive
				MYOCD			
#3 24 hour	1	0	1	13	15		7%
#3+AIP 24 hour	7	3	0	4	14	**0.003**	50%
#3+Cont. 24 hour	0	6	0	7	13	1.000	0%
#3 48 hour	0	3	1	11	15		0%
#3+AIP 48 hour	5	3	0	0	8	**0.0002**	63%
#3+Cont. 48 hour	0	4	0	5	9	1.000	0%
				NKX2.5			
#3 24 hour	0	0	0	8	8		0%
#3+AIP 24 hour	8	1	0	1	10	**0.0004**	80%
#3+Cont. 24 hour	0	1	0	8	9	1.000	0%
#3 48 hour	0	0	1	5	6		0%
#3+AIP 48 hour	12	0	1	0	13	**0.0003**	92%
#3+Cont. 48 hour	3	2	3	2	10	0.209	30%
				VMHC1/MYH15			
#3 24 hour	0	0	0	7	7		0%
#3+AIP 24 hour	1	4	0	6	11	1.000	9%
#3+Cont. 24 hour	0	0	0	7	7	1.000	0%
#3 48 hour	0	0	0	10	10		0%
#3+AIP 48 hour	5	2	0	1	8	**0.001**	63%
#3+Cont. 48 hour	0	1	0	7	8	1.000	0%
				IRX4			
#3 24 hour	0	0	0	8	8		0%
#3+AIP 24 hour	0	2	1	3	6	1.000	0%
#3+Cont. 24 hour	0	0	0	6	6	1.000	0%
#3 48 hour	0	0	0	7	7		0%
#3+AIP 48 hour	7	2	4	2	15	**0.045**	47%
#3+Cont. 48 hour	0	1	0	13	14	1.000	0%
				NPPB			
#3 48 hour	0	0	0	8	8		0%
#3+AIP 48 hour	1	5	2	2	10	**0.034 †**	60% †
#3+Cont. 48 hour	0	0	0	10	10	1.000	0%
				GJA5			
#3 48 hour	0	0	0	7	7		0%
#3+AIP 48 hour	5	2	0	2	9	**0.028**	56%
#3+Cont. 48 hour	0	2	0	6	8	1.000	0%
				SHOX2			
#3 48 hour	0	0	1	6	7		0%
#3+AIP 48 hour	2	1	0	5	8	0.462	25%
#3+Cont. 48 hour	0	0	1	7	8	1.000	0%
				AMHC1			
#3 24 hour	0	0	0	14	14		0%
#3+AIP 24 hour	0	0	2	7	9	1.000	0%
#3+Cont. 24 hour	0	0	2	7	9	1.000	0%
#3 48 hour	0	0	1	7	8		0%
#3+AIP 48 hour	2	1	1	4	8	0.200	25%
#3+Cont. 48 hour	0	1	0	7	8	1.000	0%
#1 48 hour	8	2	1	1	12		67%
#1+AIP 48 hour	4	4	0	8	16	**0.043**	25%
#3+Cont. 48 hour	6	2	1	1	10	1.000	60%

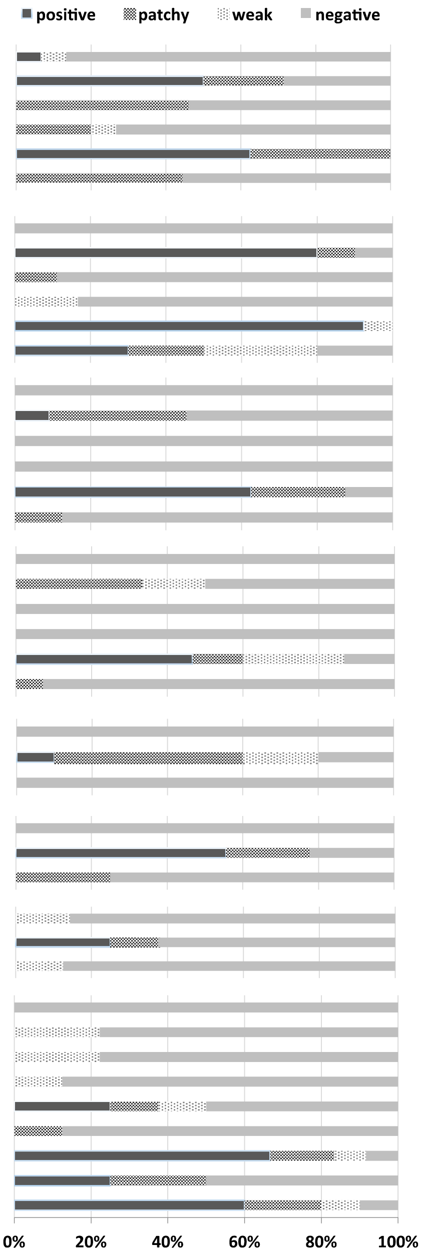

Explants of #3-mesoderm cultured in vitro are scored as positive for expression of *MYOCD*, *NKX2.5*, *VMHC1/MYH15*, *IRX4* and *GJA5* and patchy *NPPB* but not *AMHC1* or *SHOX2* when co-cultured with AIP for 24 or 48 hours. Cardiac markers are not induced in #3-mesoderm when co-cultured with control (Cont.), non-AIP endoderm. Explants of #1-mesoderm cultured in vitro are scored as positive for expression of *AMHC1*. Explants of #1-mesoderm are scored as negative for expression of *AMHC1* when co-cultured with AIP for 48 hours but not by co-culture with control (Cont.), non-AIP endoderm. † patchy expression included in the *p*-value calculation. Percentage stacked histograms are depicted for each condition.

Supplementary Table 4. Anterior-lateral endoderm does not induce ventricular genes in non-cardiac mesoderm.

	positive	patchy	weak	negative	total	p-value	% positive
	VMHC1/MYH15						
#3	0	0	0	6	6		0%
#3+#1-2 Endo	2	0	3	10	15	1.000	13%
#3+#3 Endo	1	0	1	6	8	1.000	13%
	IRX4						
#3	0	0	0	6	6		0%
#3+#1-2 Endo	1	0	1	11	13	1.000	8%
#3+#3 Endo	0	0	0	7	7	1.000	0%
	NPPB						
#3	1	0	1	4	6		17%
#3+#1-2 Endo	3	1	2	8	14	1.000	21%
#3+#3 Endo	1	0	1	3	5	1.000	20%
	GJA5						
#3	0	0	0	6	6		0%
#3+#1-2 Endo	0	3	3	8	14	1.000	0%
#3+#3 Endo	3	1	1	2	7	0.182	43%
	AMHC1						
#3	0	0	0	7	7		0%
#3+#1-2 Endo	1	2	2	6	11	1.000	9%
#3+#3 Endo	1	0	2	2	5	0.417	20%

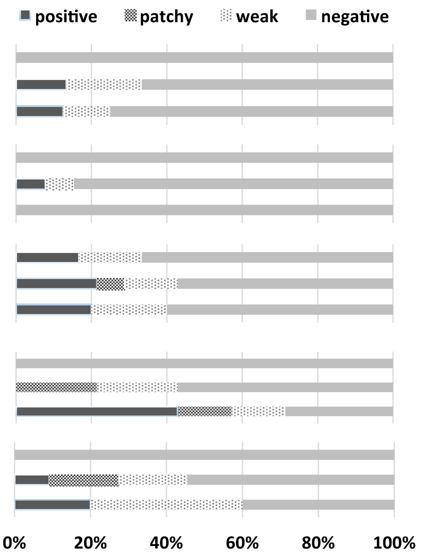

VMHC1/MYH15, IRX4, NPPB, *GJA5* or *AMHC1* are not induced in #3-mesoderm when co-cultured with anterior-lateral endoderm (#1-2E) in a host chick embryo. Cardiac markers are not induced in #3-mesoderm when co-cultured with control, anterior-medial endoderm (#3E). Percentage stacked histograms are depicted for each condition.

Supplementary Table 5. Organizers are partially interchangeable.

	positive	patchy	weak	negative	total	p-value	% positive
				MYOCD			
#3	0	0	0	7	7		0%
#3+HN	0	0	0	3	3	1.000	0%
#3+PS	0	0	0	3	3	1.000	0%
#3 24 hour	1	0	1	13	15		7%
#3+HN 24 hour	0	0	2	5	7	1.000	0%
#3+PS 24 hour	0	1	0	4	5	1.000	0%
				VMHC1/MYH15			
#3	0	0	0	6	6		0%
#3+HN	0	2	0	3	5	0.182 †	40% †
#3+PS	0	0	0	5	5	1.000	0%
#3 48 hour	0	0	0	10	10		0%
#3+HN 48 hour	2	3	0	4	9	**0.011 †**	56% †
#3+PS 48 hour	1	0	0	6	7	0.412	14%
				SOX3			
Cont.	0	0	0	4	4		0%
AIP	4	0	0	0	4	**0.029**	100%
				SOX2			
Cont.	0	0	0	4	4		0%
AIP	0	0	0	4	4	1.000	0%

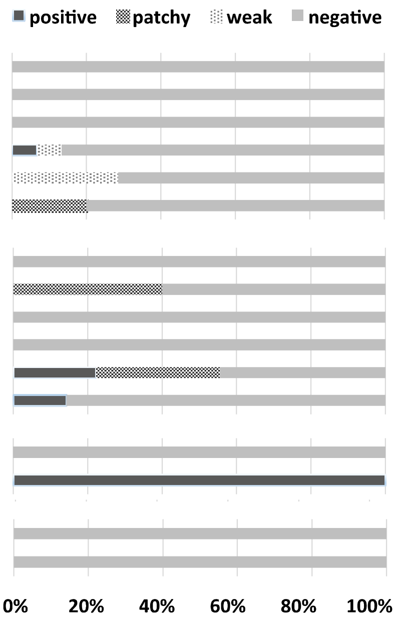

Hensen's Node (HN) induces patchy expression of *VMHC1/MYH15* but not *MYOCD* in explants of #3-mesoderm when co-cultured either in a host chick embryo or in vitro (grey). *VMHC1/MYH15* and *MYOCD* are not induced in #3-mesoderm when co-cultured with posterior primitive streak (PS). AIP induces *SOX3* in the epiblast of a host embryo. † patchy expression included in the *p*-value calculation. Percentage stacked histograms are depicted for each condition.

Supplementary Table 6. Secreted molecules induce *VMHC/MYH15* and *NPPB* in non-cardiac mesoderm.

	positive	patchy	weak	negative	total	p-value	% positive/patchy
				MYOCD			
#3	0	0	0	7	7		0%
#3 + pCAB	0	0	0	14	14	1.000	0%
#3 + NRP1+FBLN7+KIRREL3+VTN	0	0	0	7	7	1.000	0%
				NKX2.5			
#3	0	0	0	6	6		0%
#3 + NRP1+FBLN7+KIRREL3+VTN	0	0	0	8	8	1.000	0%
#3 + pCAB	0	0	0	8	8	1.000	0%
				GATA4			
#3	0	0	0	6	6		0%
#3 + NRP1+FBLN7+KIRREL3+VTN	0	0	0	5	5	1.000	0%
#3 + pCAB	0	0	0	5	5	1.000	0%
				TBX5			
#3	0	0	0	7	7		0%
#3 + NRP1+FBLN7+KIRREL3+VTN	0	0	0	5	5	1.000	0%
#3 + pCAB	0	0	0	5	5	1.000	0%
				ISL1			
#3	0	0	0	6	6		0%
#3 + NRP1+FBLN7+KIRREL3+VTN	0	0	0	5	5	1.000	0%
#3 + pCAB	0	0	0	5	5	1.000	0%
				MEF2C			
#3	0	0	0	5	5		0%
#3 + NRP1+FBLN7+KIRREL3+VTN	0	0	0	5	5	1.000	0%
#3 + pCAB	0	0	0	5	5	1.000	0%
				VMHC1/MYH15			
#3	0	0	0	6	6		0%
#3 + pCAB	1	0	0	33	34	1.000	0%
#3 + NRP1+FBLN7+KIRREL3+VTN	0	3	0	7	10	0.250 †	30% †
				IRX4			
#3	0	0	0	6	6		0%
#3 + NRP1+FBLN7+KIRREL3+VTN	0	0	0	7	7	1.000	0%
#3 + pCAB	0	0	0	7	7	1.000	0%
				NPPB			
#3	1	0	1	4	6		17%
#3 + pCAB	0	0	0	10	10	0.375	0%
#3 + NRP1+FBLN7+KIRREL3+VTN	0	2	0	8	10	0.474 †	20% †

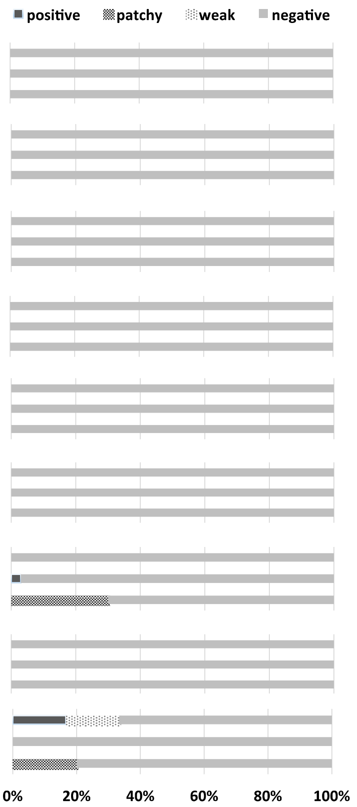

Explants of #3-mesoderm cultured in a host embryo are scored as patchy for expression of *VMHC1/MYH15* and *NPPB* when co-cultured with cell pellets transfected with NRP1+FBLN7+KIRREL3+VTN and negative for expression of *MYOCD, NKX2.5*, *GATA4*, *TBX5*, *ISL1*, *MEF2C* and *IRX4*. Cardiac markers are not induced in #3-mesoderm when co-cultured with control pellets (pCAB). † patchy expression included in the *p*-value calculation. Percentage stacked histograms are depicted for each condition.
